# Pyro-layered heterostructured nanosheet membrane for hydrogen separation

**DOI:** 10.1038/s41467-023-37932-9

**Published:** 2023-04-15

**Authors:** Ruoxin Wang, Jianhao Qian, Xiaofang Chen, Ze-Xian Low, Yu Chen, Hongyu Ma, Heng-An Wu, Cara M. Doherty, Durga Acharya, Zongli Xie, Matthew R. Hill, Wei Shen, Fengchao Wang, Huanting Wang

**Affiliations:** 1 Department of Chemical and Biological Engineering, Monash University, Clayton, Victoria 3800 Australia; 2 CAS Key Laboratory of Mechanical Behavior and Design of Materials, Department of Modern Mechanics, University of Science and Technology of China, Hefei, 230027 China; 3 School of Chemistry and Molecular Engineering, East China Normal University, Shanghai, 200241 China; 4 State Key Laboratory of Materials-Oriented Chemical Engineering, National Engineering Research Center for Special Separation Membrane, Nanjing Tech University, Nanjing, 210009 China; 5 Monash Center for Electron Microscopy, Monash University, Clayton, Victoria 3800 Australia; 6 CSIRO Manufacturing, Private Bag 10, Clayton South, Victoria 3169 Australia

**Keywords:** Chemical engineering, Two-dimensional materials

## Abstract

Engineering different two-dimensional materials into heterostructured membranes with unique physiochemical properties and molecular sieving channels offers an effective way to design membranes for fast and selective gas molecule transport. Here we develop a simple and versatile pyro-layering approach to fabricate heterostructured membranes from boron nitride nanosheets as the main scaffold and graphene nanosheets derived from a chitosan precursor as the filler. The rearrangement of the graphene nanosheets adjoining the boron nitride nanosheets during the pyro-layering treatment forms precise in-plane slit-like nanochannels and a plane-to-plane spacing of ~3.0 Å, thereby endowing specific gas transport pathways for selective hydrogen transport. The heterostructured membrane shows a high H_2_ permeability of 849 Barrer, with a H_2_/CO_2_ selectivity of 290. This facile and scalable technique holds great promise for the fabrication of heterostructures as next-generation membranes for enhancing the efficiency of gas separation and purification processes.

## Abstract (web summary)

Engineering 2D heterostructures with unique physiochemical properties and molecular sieving channels is one approach for designing membranes selective gas molecule transport. Here authors arrange graphene and boron nitride nanosheets in an alternating pattern, resulting in narrow porous nanochannels and excellent hydrogen separation properties.

## Introduction

Membrane-based hydrogen separation technologies have advantages over conventional processes^1,2^ in energy efficiency, capital cost, and footprint^3–5^. However, current commercial membranes, which are fabricated from a few polymeric materials, suffer from the trade-off between gas permeability and selectivity^6^. Laminar membranes fabricated from two-dimensional (2D) nanosheets^7,8^, such as graphene oxide (GO)^9–12^, MXene^13,14^ and molybdenum disulfide (MoS_2_)^15^, can outperform the current gas permeability-selectivity upper bound by enabling molecular transport through in-plane channels and plane-to-plane interlayer spacings^11,13^. Nonetheless, engineering the interlayer spacing between neighboring nanosheets as gas transport pathways is crucial to fast and selective molecular transport^13,16–19^. Various techniques have been proposed to regulate gas transport pathways, including chemical cross-linking^12,20–22^, mechanical compression^11^, and pore etching^23,24^. Yang and coworkers developed a thiourea covalently linked GO framework (TU-GOF) membrane with narrow and well-defined 2D channels of 3.7 Å due to the small linkers and the interaction between GO and TU^20^. Shen and coworkers applied an external force on a GO laminate to direct GO nanosheet stacking, realizing subnanometer 2D apertures with an interlayer height of 4.0 Å^11^. However, the random arrangement of nanosheets causes nonselective in-plane defects and plane-to-plane spacing when they are stacked into membranes^11,13^. It is very important to develop effective strategies for not only precise manipulation of the gas transport pathways but also for minimization of the defects caused by the random stacking of 2D nanosheets.

Recently, fabricating van der Waals heterostructures by stacking different 2D materials alternatingly^25–28^ or by chemical growth techniques^26,29,30^ has shown unique synergistic physicochemical properties, revealing great opportunities in tunneling and photovoltaic applications^26^. Charge redistribution might occur between the adjoining 2D laminate, resulting in manipulated electronic or ionic states in each layer, which further helps the adsorption and diffusion of ions. Such stacking can also induce changes in each structure, which can be finely controlled by adjusting the relative orientation^26^. Thus, heterostructures with an extended range of functionalities possess an increasing number of possible applications.

**Fig. 1:**
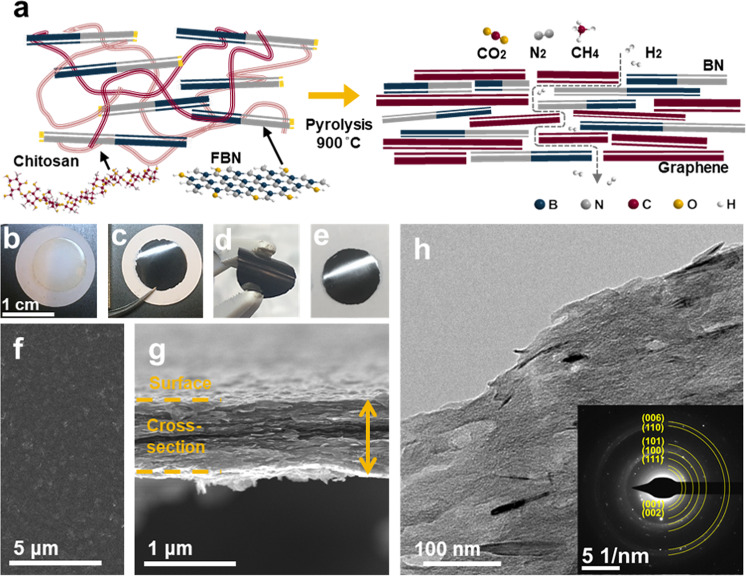
Preparation, morphology and structure of boron nitride and graphene nanosheet (BNG) heterostructured membrane. **a** Schematic illustration depicting the pyro-layering approach and gas molecule transport through the BNG membrane. **b**, **c** Digital images of the prepared FBN-chitosan precursor membrane and BNG membrane before and after high-temperature pyro-layering. **d**, **e** Digital images of a free-standing BNG membrane bent by tweezers and placed on the bench. **f** SEM surface-view image of the BNG membrane. **g** SEM cross-sectional image of the BNG membrane. **h** TEM image of a cross-section of the BNG membrane. The inset represents the selected area electron diffraction (SAED) patterns of the BNG membrane. All precursor and BNG membranes with 53.2% BN shown in Fig. 1 were prepared with an FBN-chitosan precursor solution with the same ratio (FBN: chitosan = 7:10).

Herein, we prepare a 2D nanosheet-based heterostructured molecular sieving membrane through a high-temperature pyro-layering approach. We use boron nitride nanosheets as the main scaffold and graphene nanosheets derived from the chitosan precursor as the filler in creating the heterostructure. The rearrangement of graphene-like nanosheets adjoining the boron nitride nanosheets^31,32^ forms precise nanochannels for highly selective H_2_ transport (Fig. 1a). The resulting boron nitride and graphene nanosheet (BNG) heterostructured membrane possesses unique gas transport pathways for fast and selective H_2_ transport that surpass most current state-of-the-art gas separation membranes.

## Results

### Fabrication and Characterization of Boron Nitride and Graphene Heterostructured Membranes

The BNG membranes were prepared via a two-step pyro-layering procedure (Supplementary Fig. 1) from functionalized boron nitride nanosheets (FBN) and chitosan precursors (Supplementary Figs. 2–5). FBN was first intercalated with chitosan in the preparation of the precursor solution, followed by vacuum filtration of the FBN-chitosan mixture on porous alumina substrates. The FBN-chitosan precursor membrane was subsequently heat-treated in an argon atmosphere at high temperature to carbonize chitosan to graphitic carbon sheets (graphene). Figure 1a shows the pyro-layering formation of a boron nitride (BN)-graphene nanosheet heterostructured membrane consisting of alternatingly stacked boron nitride nanosheets (BN) and chitosan-derived graphene as well as the gas permeation pathway of the in-plane and plane-to-plane nanochannels.

As shown in Figs. 1b and 1c, the appearance of the as-prepared membrane turns from semitransparent yellow to metallic black after pyro-layering. The BNG membrane can be readily peeled off from the alumina substrate while maintaining high mechanical integrity and flexibility (Figs. 1d and 1e). For comparison, pure FBN and carbonized chitosan films were also prepared. Both pure FBN and carbonized chitosan films are brittle, showing semitransparent white and metallic black colors, respectively (Supplementary Fig. 4). In the case of the pure FBN film, the vacuum filtration process of rigid FBN leads to random stacking, resulting in pinholes on the surface (Supplementary Fig. 5a). On the other hand, FBN-chitosan precursor membranes exhibit a smooth and defect-free surface morphology with a homogenous distribution of FBN (Supplementary Fig. 6). Chitosan-derived graphene occupies the space between the adjoining nanosheets after the pyro-layering process (Supplementary Fig. 7). The scanning electron microscopy (SEM) images of the membrane surface (Fig. 1f and Supplementary Fig. 8a, b, d and e) reveal a dense morphology without visible defects or cracks on the fabricated BNG membranes. In addition, the BN and graphene sheets are integrated to form the BNG membranes, as the BN is uniformly distributed in the heterostructure layers without aggregation (Fig. 1f and Supplementary Fig. 8). The cross-sectional SEM image of the BNG membrane (Fig. 1g) shows a uniform thickness of ~ 800 nm with horizontally ordered and well-packed BN. The BNG membrane prepared with a precursor solution of FBN:chitosan = 7:25 shows a membrane thickness of ~ 850 nm (Supplementary Fig. 8c), and the BNG membrane prepared with a ratio of FBN:chitosan = 7:5 (Supplementary Fig. 8f) has a thickness of ~ 900 nm. SEM images (Fig. 1f, g, Supplementary Fig. 8) indicate that the BN and graphene heterostructures with different ratios are uniformly stacked by the pyro-layering strategy, effectively eliminating any random stacking or agglomeration by the filtration process. The selected area electron diffraction (SAED) pattern indicates that the BNG membrane is polycrystalline^33^, with several characteristic diffraction rings of boron nitride and graphene heterostructure (Fig. 1h, inset).

**Fig. 2:**
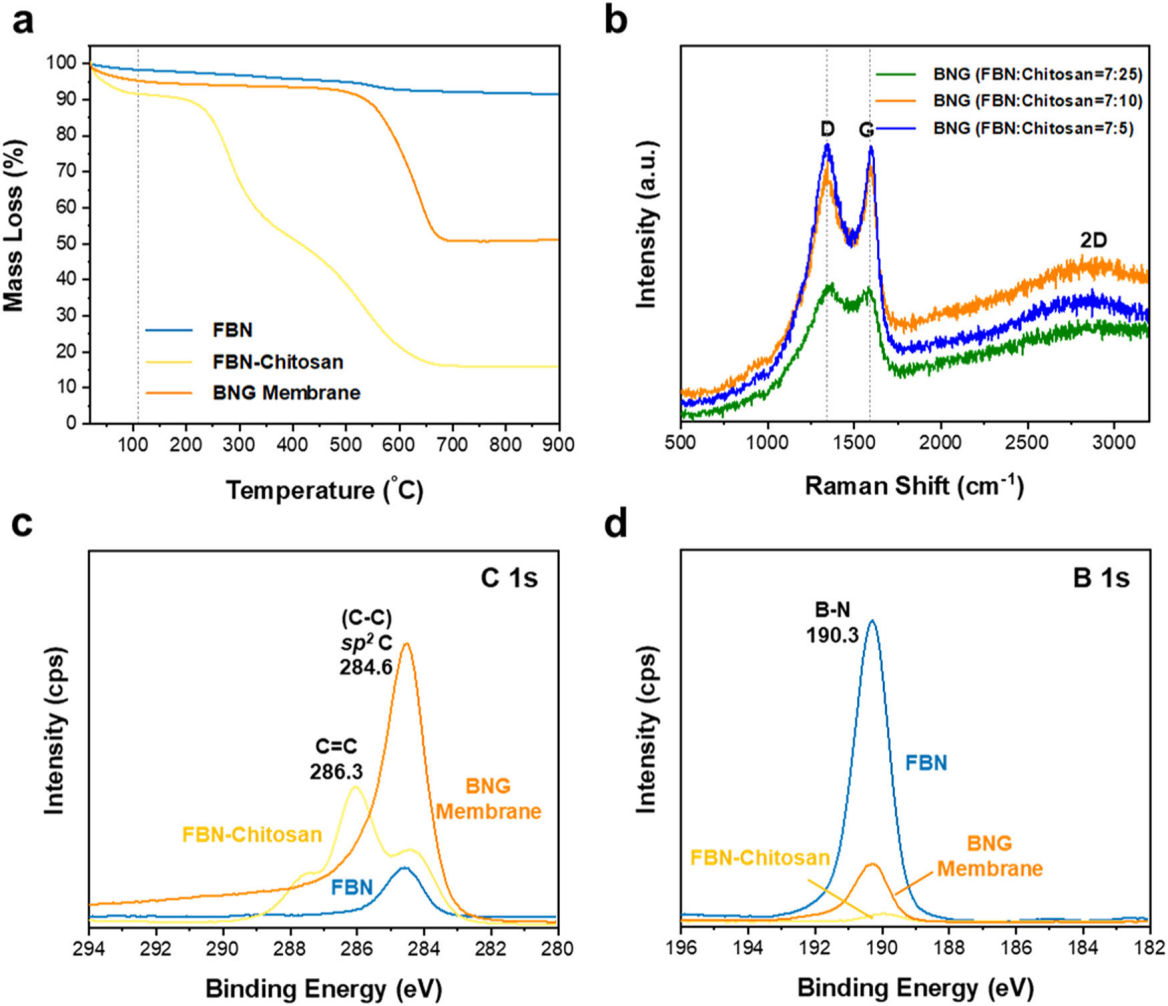
Chemical structure of FBN and chitosan films, FBN-chitosan precursor and the prepared BNG heterostructured membranes. **a** Thermogravimetric analysis (TGA) of the FBN film, FBN-chitosan precursor and BNG membranes (FBN:chitosan = 7:10). **b** Raman spectra of BNG membranes with different ratios (FBN:chitosan = 7:25, 7:10, and 7:5). **c**, **d** XPS spectra of the chemical states of the elements (C 1 s and B 1 s) on the surface of the BNG membrane (FBN:chitosan = 7:10).

TGA analysis (Fig. 2a) reveals that the actual mass percentage of BN in the heterostructured membrane accounts for approximately 53.2%, which is much higher than that in the precursor solution (41.2 %) and FBN-chitosan precursor membrane (approximately 17.50 %) (detailed calculation in the Supplementary Notes). The mass loss that occurred at approximately 110 °C is attributed to the desorption of the absorbed moisture from the air (indicated as a gray dashed line). Raman spectroscopy was used to analyze the graphitic carbon species in the heterostructure (Fig. 2b). The BNG membranes show the characteristic G peak at 1590 cm^−1^, which can be ascribed to the sp^2^ amorphous carbon atom. The 2D peak at 2700 cm^−1^ demonstrates the existence of graphene, while the distinct D peak at 1350 cm^−1^ shows the intensity of the defects or edges from graphene^34,35^. In addition, with increasing FBN loading, the increased intensity of the G and D peaks suggests that more defects exist in the BNG membranes^33^. In contrast, FBN only shows the characteristic peak at approximately 1381 cm^−1^ (Supplementary Fig. 2c). Furthermore, the X-ray photoelectron spectrum (XPS) of C1 s (Fig. 2c) shows that the BNG membrane has a characteristic peak at 284.60 eV, which can be attributed to the formation of typical sp^2^ carbon atoms after pyro-layering. For the B 1 s and N 1 s spectra, the two peaks with binding energies at 190.30 and 397.95 eV (Fig. 2d and Supplementary Fig. 9e) confirm the existence of B-N bonds of BN in all samples.

### Characterization of nanochannels

**Fig. 3:**
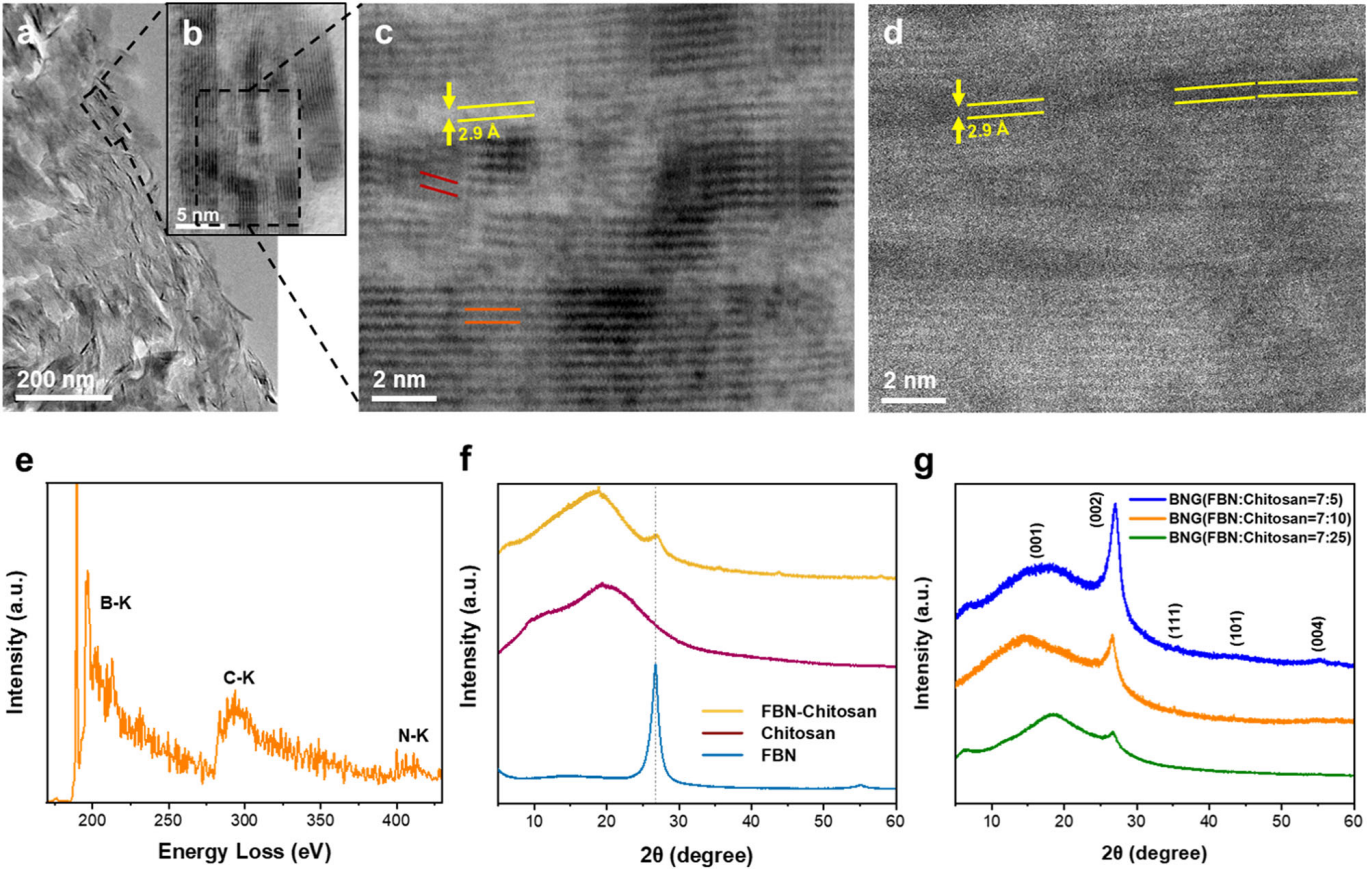
Analysis of the nanostructure of BNG membranes. **a** TEM image of the BNG membrane cross-section (FBN: chitosan = 7:10). **b** HRTEM image of the BNG membrane cross-section in the selected area shown in (**a**). **c** HRTEM image of the cross-section of the BNG membrane in the selected area shown in (**b**). The HRTEM image reveals the alternating stacking of BN and graphene sheets. Interlayer spacings of graphene and BN sheets are illustrated in red and orange, respectively, and the width of the formed nanochannel is indicated in yellow. **d** HAADF-HRTEM image of the same selected area shown in (**c**). **e** Electron energy-loss spectra of the B K-edge, C K-edge, and N K-edge of the BNG membrane (FBN: chitosan = 7:10). **f** XRD patterns of pure FBN and chitosan films and the FBN-chitosan precursor (FBN:chitosan = 7:10). **g** XRD patterns of the BNG membranes (FBN: chitosan = 7:25, 7:10 and 7:5).

As shown in Fig. 3a, the transmission electron microscopy (TEM) image of the BNG membrane cross-section shows some laminar domains, whose lengths are consistent with those of FBN (Supplementary Fig. 3a). When analyzed with high-resolution transmission electron microscopy (HRTEM) of the cross-section of the BNG membrane in the selected area, the ordered layers indicate that the BN and graphene sheets are stacked in the same direction (Fig. 3b). Moreover, Fig. 3b shows a stacked morphology of the layered nanosheets, which is consistent with few-layered FBN (Supplementary Fig. 3b). The interfaces between the stacked BN and graphene can be observed in Fig. 3c. The few-layer nanostructure and the interlayer spacing (≈3.3 Å) between the BN (indicated by the orange lines) are consistent with those of the FBN shown in Supplementary Fig. 3b. The derived graphene sheets (indicated by black lines), on the other hand, seamlessly wrap around the BN. Graphene nanosheets alongside BN illustrate similar stacking arrangements, while the graphene at the edges of BN forms labyrinth-like patterns (Supplementary Fig. 10). The interlayer spacing of 3.4 Å is consistent with the interlayer spacing between the graphene sheets^36,37^. As expected, chitosan-derived graphene fills the vacancy between the neighboring BN. By inserting chitosan-derived graphene sheets, the width of the formed nanochannel was measured to be 2.9 Å. During the assembly of chitosan with FBN in precursor membrane preparation, chitosan molecules are likely to deposit at the edges and defects of FBN with oxygen functional groups^38^. The pyro-layering process facilitates chitosan carbonization to form a graphitic carbon (few-layer graphene) nanostructure^39,40^. Therefore, the preparation of the heterostructured membrane demonstrates that the pyro-layering approach is effective for precise control of the 2D channels and repair of the defects between the stacked nanosheets. As shown in Fig. 3d, the high-magnification high-angle annular dark-field scanning transmission electron microscopy (HAADF-STEM) image from the same membrane cross-section (Fig. 3c) reveals the distinct nanochannels formed by derived graphene and adjoining BN nanosheets (indicated by the yellow lines). To further analyze the composition of the heterostructure, electron energy loss spectroscopy (EELS) (Fig. 3e) was used to identify the mass density of single atoms. Boron (B), carbon (C), and nitrogen (N) K-edge spectra at approximately 200, 295, and 410 eV can be observed^41–43^. The B atom shows a higher intensity than the C atom, suggesting a higher mass content in the heterostructure layer. The narrow peak at approximately 285 eV shows the relative component of sp^2^ orbitals within the sample and indicates the existence of amorphous carbon in the BNG heterostructure membrane^44^.

As shown in the X-ray diffraction (XRD) patterns in Fig. 3f, g, all BN-related samples except for the pure chitosan film exhibit a prominent peak at approximately 27°, corresponding to an interlayer spacing of ~ 3 Å. Compared with the FBN-chitosan precursor membrane, the increasing intensity at 27° indicates the homogeneous distribution of BN in the BNG heterostructured layer after pyro-layering. The XRD pattern of the BNG membrane with 53.2% BN exhibits another peak at 14.3°, translating to an interlayer spacing of ~6.2 Å (Fig. 3g)^38^. The interlayer height between the neighboring sheets can be estimated to be 2.8 Å by taking the thickness of the monolayer graphene (~3.4 Å) into account^45^. The two characteristic diffraction peaks at ~14.3° and 27° arise from the (001) and (002) planes of the BNG heterostructure, which is consistent with the SAED image (Fig. 1h, inset).

To determine the pore size distribution of the BNG membrane, the nitrogen sorption technique was initially used, but the membrane had a low Brunauer‒Emmett‒Teller (BET) surface area of 21 to 28 m^2^/g, making it difficult to calculate its pore size distribution from the isotherm. Then, the membrane sample was analyzed using CO_2_ physisorption at 0 °C^34^. As shown in Supplementary Fig. 11), the BNG membrane shows a pore size distribution of 3–10 Å with a peak pore width of 3.4 Å, which is slightly greater than the nanochannel height obtained by the HRTEM images (Figs. 3c and 3d) and XRD results (Fig. 3g). The total pore volume is 0.036 cm^3^/g, corresponding to a Brunauer‒Emmett‒Teller (BET) surface area of 100.6 m^2^/g. Furthermore, the XRD patterns of the BNG membranes (FBN:chitosan = 7:25 and 7:5) show peaks at 16.10° and 15.05°, translating to interlayer spacings of ~5.5 and 5.9 Å, respectively, which are smaller than those of the BNG membrane prepared with FBN:chitosan = 7:10. The characteristic peak of BN at 27° becomes stronger at a higher loading of BN in the heterostructured membranes. The FBN-chitosan precursor membrane and pure chitosan film exhibit characteristic broad peaks at 19° and 19.5°, indicating amorphous structures (Fig. 3f). The slight change in the peak intensity can be attributed to the embedment of FBN in the chitosan matrix^35^.

Positron annihilation lifetime spectroscopy (PALS) was performed to further analyze the membrane microporosity. The average pore diameter of the BNG membrane from PALS is 3.72 ± 0.14 Å (Supplementary Table 1), which is slightly larger than the results obtained by the HRTEM image (Fig. 3c), XRD analysis (Fig. 3g) and CO_2_ sorption technique (Supplementary Fig. 11). The larger average pore of 3.72 Å is likely due to the model being designed for a spherical pore model rather than nanosheets^46^. Small pores on the film surface can be observed from the SEM image of the carbonized chitosan (Supplementary Fig. 7c), while all the BNG membranes show a dense morphology, suggesting that the heterostructure efficiently enhances the membrane nanostructure and reduces the inner defects (Fig. 1g, Supplementary Fig. 8c, f).

### Gas molecular sieving performance

**Fig. 4:**
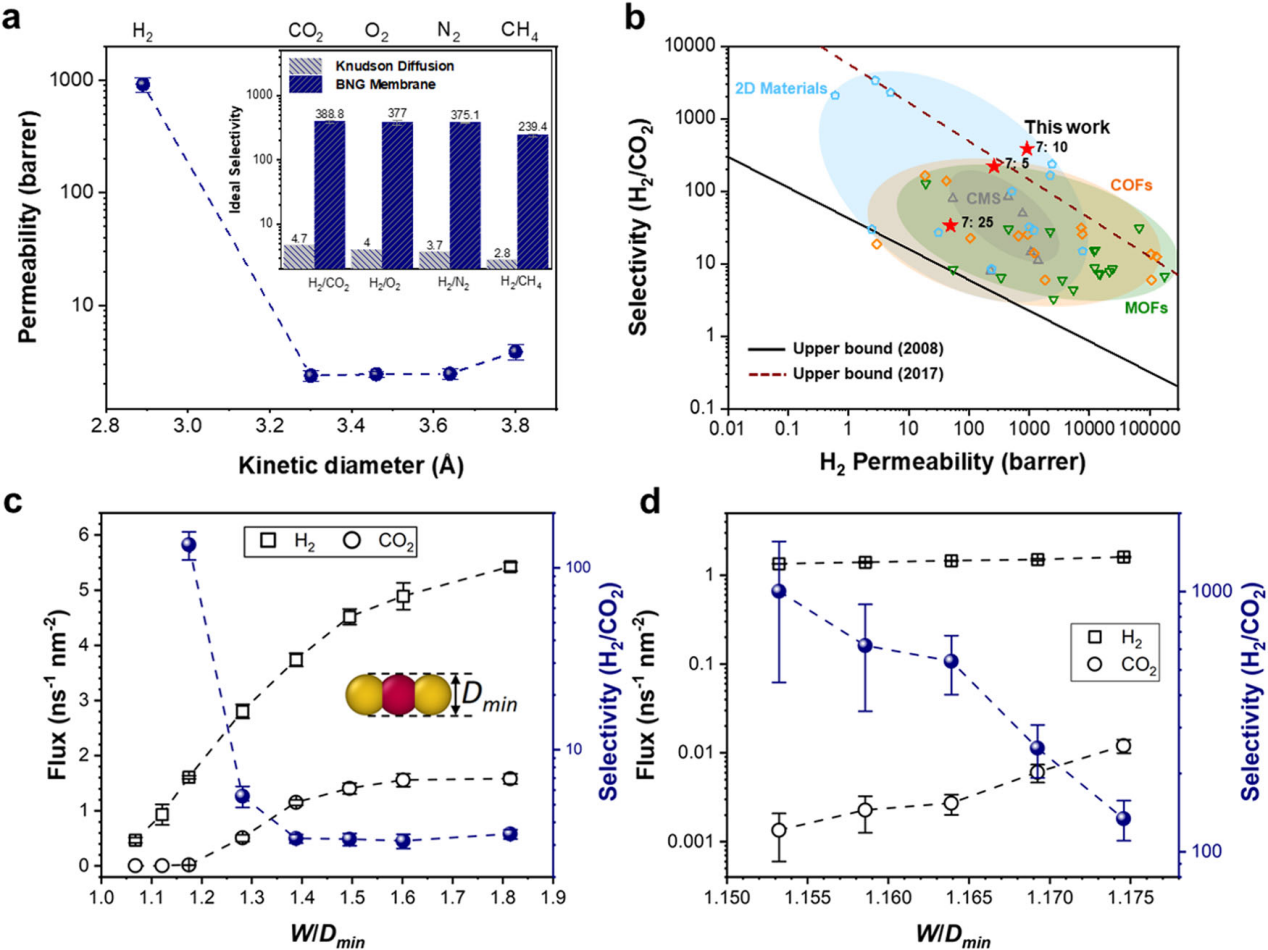
Gas separation performance and molecular dynamics simulations of the fabricated BNG heterostructured membrane (FBN:chitosan = 7:10). **a** Single gas permeability of the BNG membrane as a function of the gas kinetic diameter at 25 °C. The inset represents the ideal selectivity of hydrogen to other gases. **b** The H_2_/CO_2_ separation performance of our BNG membranes compared with other molecular sieving membranes. The black line indicates the 2008 Robeson upper bound of polymeric membranes^6^, and the burgundy dashed line indicates the 2017 upper bound of the best current membranes for H_2_/CO_2_ separation. The gas separation performance of the literature data is provided in Supplementary Table 2. **c** The dependence of gas flux and H_2_/CO_2_ selectivity on the slit width relative to the shortest dimension of CO_2_. Inset, the illustration of shortest dimension measurement. **d** A zoomed-in view of gas flux and selectivity for the slit with a width approaching the shortest dimension of CO_2_.

The gas molecular sieving performance of the BNG membranes was evaluated by measuring the single gas permeation using H_2_ (kinetic diameter: 2.89 Å), CO_2_ (3.30 Å), N_2_ (3.60 Å) and CH_4_ (3.80 Å) as the feed in an inhouse permeation apparatus at room temperature and 1 bar (Supplementary Fig. 12). Figure 4a shows a sharp cutoff of the gas permeability between H_2_ and other large gas molecules, suggesting a clear gas molecular-sieving property. The BNG membrane exhibits an H_2_ permeability of 920 Barrer but much lower CO_2_, N_2_, O_2_, and CH_4_ permeabilities. The best performing BNG membrane, at an FBN:chitosan ratio of 7:10, shows an ideal selectivity of 388 for H_2_/CO_2_, 375 for H_2_/N_2_, and 239 for H_2_/CH_4_ (Fig. 4a, inset), which are significantly over the Knudsen diffusion selectivity (4.7, 3.7 and 2.8, respectively). In contrast, the porous alumina substrate shows high H_2_ permeance and ideal selectivity of 3.7 for H_2_/CO_2_, 3.1 for H_2_/N_2_, and 2.6 for H_2_/CH_4_ (Supplementary Fig. 13). The performance of the BNG membrane was also determined using a binary gas mixture (50:50 v%) of H_2_ and CO_2_. The hydrogen selectivity of the H_2_/CO_2_ mixture was 290, which was 25% lower than the ideal selectivity. The H_2_ permeability was calculated to be 849 Barrer, which is slightly lower than that obtained using the single gas permeation experiment. A similar trend can be observed from the H_2_ permeability of the other two BNG membranes with different FBN:chitosan ratios. The decrease may be the result of the competitive diffusion or adsorption of mixed gases, which was also observed in other 2D material-based membranes for gas sieving^11,13,20^. The separation performance suggests a precise molecular sieving effect by discriminating gas molecules larger than CO_2_ (3.30 Å), which is consistent with the interlayer spacing of BNG membranes. The CH_4_ permeability is slightly higher than that of the other gas molecules except for H_2_, although its kinetic diameter is the largest (Fig. 4a). A similar trend was observed in studies investigating pristine graphene flakes and GO nanosheets for gas molecule sieving^9,46^.

The optimized BNG membranes surpass the Robeson upper bound (2008)^6^ for H_2_/CO_2_ (Fig. 4b), H_2_/N_2_ (Supplementary Fig. 14a) and H_2_/CH_4_ (Supplementary Fig. 14b) separation as a function of hydrogen permeability (Supplementary Tables 2–4). As shown in Fig. 4b, the BNG membrane demonstrates superior performance to porous material-assembled membranes, such as metal-organic frameworks (MOFs), covalent-organic frameworks (COFs) and zeolites. Compared with other 2D material-based membranes, the BNG membrane exhibits promising H_2_ separation properties because the larger gas molecules are more precisely discriminated by the slit-like in-plane and plane-to-plane nanochannels throughout the heterostructured membrane.

To optimize the H_2_ separation performance, BNG membranes with different FBN:chitosan ratios were prepared under the same pyro-layering process while maintaining similar membrane thicknesses (Fig. 1g and Supplementary Fig. 8c, f). The single gas permeability and ideal and mixed gas selectivity are summarized in Supplementary Table 5. The difference in gas molecular sieving performance may be ascribed to the number of constructed pathways by the alternating arrangement of BN and hybridized amorphous graphene nanosheets (as illustrated in Fig. 1a). BN acts as the scaffold of the heterostructure, while the hybridized graphene nanosheets adjoining the BN fill the defects. The formation of precisely tuned nanochannels between neighboring nanosheets at optimal ratios provides an ideal gas transport pathway for H_2_ molecules. Uniform and small-sized BN throughout the heterostructure increases the molecular pathways, leading to a high hydrogen permeance^47,48^. On the other hand, the excessive carbon content in the heterostructured membrane may reduce the formation of interconnected nanochannels, resulting in low hydrogen permeability. Thus, as shown in Fig. 4b, the BNG membrane, at an FBN:chitosan ratio of 7:25, shows a performance close to that of carbon molecular sieving membranes. As shown in Supplementary Table 5, the hydrogen permeability increases sharply at a higher BN loading (FBN: chitosan = 7:10). However, the excessive loading of BN (FBN: chitosan = 7:5) leads to a slight decrease in hydrogen permeation, which can be attributed to the increased tortuosity for gas molecule transport^8,49^. In addition, the excessive loading of BN in the heterostructure led to a brittle membrane. Therefore, an optimized ratio between BN and graphene/graphite not only contributes to the high mechanical integrity of the BNG membrane but also exhibits gas separation performance exceeding most state-of-the-art membranes.

Various mechanisms play a vital role in membrane gas separation, including Knudsen diffusion^50,51^, surface diffusion^52^, adsorptive separation^53^ and molecular sieving^54^. Molecular sieving is expected to be dominant when the characteristic width of the nanochannel is close to the diameter of the gas molecules. In this work, the widths of slit-like nanochannels measured in experiments are greater than the H_2_ kinetic diameter (2.89 Å) and close to the CO_2_ kinetic diameter. Thus, molecular sieving could be the determining factor in the gas separation observed in the present work.

Molecular sieving involves a competition between the channel width and the gas molecular dimension. To explore this competition, we conducted molecular dynamics (MD) simulations calculating the flux and selectivity of H_2_/CO_2_ mixture through BN/graphene slits with varying width (Supplementary Fig. 15). Both CO_2_ and H_2_ molecules have a non-spherical appearance, and are characterized by a bar shape. When a non-spherical gas molecule attempts to enter a slit with a width comparable to its molecular dimension, the orientation of this gas molecule is a significant factor in determining whether it can enter the slit (Supplementary Fig. 16). Because the molecular projection length in the slit’s normal direction varies depending on the molecular orientation. The gas molecule can only enter the slit when the projection length is less than the slit width. There is an orientation with the shortest projection length, which we refer to as the shortest dimension (Supplementary Fig. 16). In this work, the shortest dimension is a critical criterion for gas diffusion. When the slit width is smaller than the shortest dimension, gas molecules cannot penetrate the slit. The shortest dimension of CO_2_ is greater than that of H_2_. When the channel width reduces and approaches the shortest dimension of CO_2_, CO_2_ experiences a sudden decrease in flux and finally has no permeation, while H_2_ still has a detectable flux, thereby causing a significant increase in the gas selectivity (H_2_/CO_2_) (Fig. 4c, d and Supplementary Fig. 17). Similarly, as the channel width gets closer to the shortest dimension of CH_4_, the selectivity of H_2_/CH_4_ exhibits a significant increase (Supplementary Figs. 17 and 18). High gas selectivity by molecular sieving occurs when the pore size is larger than the shortest dimension of one molecule but comparable to or even smaller than that of other molecules. Our simulation results support the notion of molecular sieving as the vital mechanism for efficient hydrogen selectivity between heterostructure nanosheets.

Moreover, we observed that CH_4_ has a higher flux than CO_2_ as the slit width increases. We attribute this to that the Knudsen diffusion mechanism comes into play, and the smaller molecular weight of CH_4_ results in a greater flow rate. This discovery may provide an explanation for why CH_4_ has a slightly higher permeability in the BNG membrane than CO_2_, even though CH_4_ has a larger kinetic diameter.

In summary, we have demonstrated a pyro-layering approach for the fabrication of robust 2D nanosheet heterostructured membranes composed of boron nitride nanosheets and graphene nanosheets derived from a chitosan precursor. Slit-like nanochannels were formed between the well-tuned nanosheets throughout the heterostructured membrane. The resultant BNG heterostructured membranes show highly preferential hydrogen molecule transport, leading to high hydrogen permeance and selectivity. The pyro-layering strategy offers an accessible and potentially scalable technique to prepare heterostructured membranes from various 2D materials not only for gas separation but also for desalination, organic solvent and ion separation.

## Methods

### Synthesis of boron nitride/graphene nanosheet heterostructured membranes (BNG heterostructured membranes)

Functionalized boron nitride nanosheets (FBN) were prepared by the mechanical method^55^, as described in our previous work^56^. The acetic acid diluted solution was prepared by diluting 0.3 mL acetic acid in 25 mL Milli-Q water and stored in a refrigerator (4 °C) for further use. Chitosan aqueous solution was prepared by dispersing 0.1 g (low-molecular-weight) chitosan powder in 4 mL prepared acetic acid solution first and then diluted by 10 mL Milli-Q water. Various volumes of 0.1 mg/mL or 1 mg/mL FBN solution, 0.4 wt. % chitosan solution (1 mL) and 0.25 wt. % glutaraldehyde solution (0.5 mL) were dispersed in 5 or 10 mL Milli-Q water and stirred for 24 h at room temperature. Taking the preparation of the FBN-chitosan precursor solution (7:10) as an example, a 1 mg/mL FBN aqueous solution (5 mL) was diluted with 10 mL Milli-Q water in the presence of 1.0 mL chitosan solution and 0.5 mL glutaraldehyde solution. A relatively low volume of glutaraldehyde was introduced into the precursor solution to enhance the cross-linking^57^. The precursor membranes were prepared by dropping a certain volume of FBN-chitosan solution on homemade alumina substrates (diameter: 13 mm) by a lab-scale vacuum filter (Supplementary Fig. 1a). Then, the filtration-made membranes were pyrolyzed at 900 °C for 3 h in a horizontal quartz tube furnace in an argon atmosphere. The heating process is illustrated in Supplementary Fig. 1b. Chitosan was carbonized and converted to graphene at high temperature^38^, while boron nitride nanosheets (BN) with high thermal resistance dominated the scaffold of the BNG membrane.

### Characterization methods

Scanning electron microscopy (SEM): The SEM images of all samples were recorded on a Magellan 400 FEG-SEM (FEI, USA) operating at an accelerating voltage of 5 kV. All mounted samples were sputter-coated with iridium. High-resolution transmission electron microscopy (HRTEM) and electron energy loss spectroscopy (EELS): HRTEM images and EELS spectroscopy were obtained on an FEI Tecnai G2 F20 S-TWIN TEM operated at an accelerating voltage of 200 kV. High-magnification high-angle annular dark-field scanning transmission electron microscopy (HAADF-STEM) images were acquired using a HAADF detector. Selected area diffraction pattern (SEAD) images were obtained on a FEI Tecnai G2 T20 transmission electron microscope operating at an accelerating voltage of 200 kV. The TEM samples were prepared by ultramicrotomy to obtain the cross-section of the fabricated membrane. More specifically, the settled samples were slightly trimmed down (25 mm/s) to obtain the cutting faces by a Leica ultra-cut S equipped with a 35° diamond blade. The cross-sectional layers were collected by TEM carbon grids from the water trough. Raman spectra: Raman spectra were recorded using a confocal micro-Raman System (Renishaw RM 2000) equipped with a near-IR diode laser at a wavelength of 782 nm (laser power: 1.15 mW and laser spot size: 1 μm). The excitation wavelength was 514 nm. All Raman spectra were collected by fine-focusing a 50× microscope objective, and the data acquisition time was 10 s. X-ray photoelectron spectroscopy (XPS): XPS was measured on a Thermo Scientific Nexsa Surface Analysis System equipped with a hemispherical analyzer. The incident radiation was monochromatic Al Kα X-rays (1486.6 eV) at 72 W (6 mA and 12 kV, 400 × 800 μm 2 spots). Survey (wide) and high-resolution (narrow) scans were recorded at analyzer pass energies of 150 and 50 eV and step sizes of 1.0 eV and 0.1 eV, respectively. Data processing was carried out using Avantage software, and the energy calibration was referenced to the mainline of C 1 s at 284.8 eV. X-ray diffraction (XRD): The XRD patterns were recorded by using a 2*θ* range of 2–60° and a scan rate of 2 °•min^−1^ at ambient temperature on a Miniflex 600 diffractometer (Rigaku, Japan) with a Cu Kα radiation source (15 mA, 40 kV). The samples were analyzed using a physisorption analyzer (Micromeritics Triflux, USA) for characterization of BET surface area, pore volume, and pore size distribution. Both nitrogen sorption at −196 °C and carbon dioxide sorption at 0 °C were performed. Prior to gas sorption measurements, the samples were degassed at 200 °C for 1440 min to ensure the removal of adsorbed gas from the micropores of the heterostructured membranes. The pore size distribution was calculated using the Horvath-Kawazon method from the carbon dioxide sorption isotherm. Positron annihilation lifetime spectroscopy (PALS, EG&G ORTEC fast-fast spectrometer): Free-standing BNG membrane and pure carbonized chitosan film were broken into pieces and stored in foil for PALS measurements. The samples were on both sides of the positron source (1.5 × 10^6^ Bq of ^22^NaCl sealed in a Mylar envelope). At least five files, each containing 1 × 10^6^ integrated counts, were recorded for every sample. Due to the conductivity of the samples, no long-lifetime annihilation events occurred, and the average pore size was estimated from the free-annihilation using tau2. Thermogravimetric analysis (TGA): TGA measurements were recorded by a TA Instrument STD 650 TGA/DSC analyzer (USA). The pristine FBN film, the precursor membrane and the carbonized heterostructure membrane (FBN: chitosan = 7:10) were packed and weighed. Then, they were heat treated (heating rate: 20 °C/min) under an air atmosphere (Instrument grade, BOC) to burn off the carbon-related content. Finally, the white residual layer (boron nitride nanosheets, as shown in Supplementary Fig. 19) was weighed. The gas flow rate was set to 100 mL/min. Gas chromatograph: mixed gas separation was tested by an Agilent 8860 with a thermal conductivity detector (TCD).

### Gas permeation experiments

The single gas permeation of the fabricated membranes was measured using a constant-volume/variable-pressure apparatus (Supplementary Fig. 12). The gas permeation rate was recorded by fixing a piece of the prepared free-standing membrane (with alumina substrate with a diameter of 13 mm) on a stainless-steel sample holder using an Agilent Torr Seal vacuum sealant. Then, it was placed inside a Pyrex tube with feed gas flowing through and connected to an MKS 628B Baratron pressure transducer and a vacuum pump.

The gas permeance experiments were performed using the steady-state gases H_2_, N_2_, CO_2_, and CH_4_ at room temperature. To achieve steady-state permeation conditions, each single gas measurement on the permeate side of the membrane was degassed in a vacuum for half an hour.

The molar flow rate of the permeating gas was calculated from the linear pressure rise, and its coefficient was calibrated using a digital flowmeter (ADM2000, Agilent, California, USA). The feed gas is supplied at room temperature and atmospheric pressure. The effective membrane area and thickness were measured by a Vernier caliper and SEM. The gas permeance, *Pi* (mol·m^−2^·s^−1^·pa^−1^), is defined by the following equation:

1 $${P}_{i}={N}_{i}/\triangle {P}_{i}\cdot A$$

where *Ni* (mol·s^−1^) is the permeate flow rate of component gas *i*, *∆Pi* (Pa) is the transmembrane pressure difference of *i*, and *A* (m^2^) is the measured membrane area (0.16 cm^−2^). The ideal selectivity *S*_*i/j*_ was calculated from the relation between the permeance of pure *i* and *j* gases.

2 $${S}_{i/j}=\frac{{P}_{i}}{{P}_{j}}$$

Mixed-gas permeation was conducted by a gas mixture with a ratio of 50:50 applied at the feed side of the BNG membrane, and the total flow rate of the feed gas was maintained at 200 mL/min (each gas at 100 mL/min) with a feed pressure at 1 bar. The gas flow was controlled by a mass flow control system (ALICAT Scientific). The sweep gas flow was constantly controlled by another mass flow meter. A gas chromatograph system (8860, Agilent) was used to analyze the composition of the permeate gases.

The mixed selectivity (α_i/j_) of two components in the mixed-gas permeation experiment was calculated by:

3 $${\alpha }_{i/j}=\frac{{y}_{i}/{y}_{j}}{{{x}_{i}/x}_{j}}$$

where *x* and *y* ate the volumetric fractions of the corresponding component in the feed and permeate side, respectively.

### MD simulation methods

The MD simulation system is composed of feed and permeate sides connected by a BN/graphene slit-like nanochannel (Supplementary Fig. 15a). The nanochannel width *W* can be adjustable, which was determined by subtracting 0.34 nm from the interlayer distance between graphene and BN nanosheets. The feed side is filled with a binary mixture of 0.5 bar H_2_ and 0.5 bar CO_2_ (or CH_4_). Under the force of pressure-driven flow, gas molecules in the feed side will travel through the nanochannel to the permeate side. During simulations, the feed side is replenished with new gas molecules, and the molecules flowing into the vacuum side are deleted to maintain constant pressure on both sides. The gas flux was given by the first derivative of the number of deleted molecules with respect to time (Supplementary Fig. 15b). We applied the periodic boundary conditions along the X and Y axes and a reflection boundary condition along the Z axis. The velocity-Verlet integrator was implemented to update the position of atoms with a 1 fs timestep. The Berendsen thermostat^58^ was employed to keep the temperature of gas molecules at 300 K. In simulations, the channel atoms are fixed. The nonbonded interactions between atoms that are within a cutoff distance of 12 Å are modeled using Lennard‒Jones (LJ) and Coulomb potentials, given by

4 $${E}_{{ij}}=4{\varepsilon }_{{ij}}\left[{\left(\frac{{\sigma }_{{ij}}}{{r}_{{ij}}}\right)}^{12}-{\left(\frac{{\sigma }_{{ij}}}{{r}_{{ij}}}\right)}^{6}\right]+\frac{{q}_{i}{q}_{j}}{4\pi {\varepsilon }_{0}{r}_{{ij}}},$$

where *ε*_*ij*_ characterizes the interaction strength, *σ*_*ij*_ represents the effective atomic diameter, *r*_*ij*_ shows the distance between two atoms, *ε*_0_ is the permittivity of the vacuum, and *q* denotes the charge. The LJ parameters among atoms of different types were calculated using the Lorentz-Berthelot mixing rule. The bond and angle energies were calculated with the following equations:

5 $${E}_{{bond}}=\frac{1}{2}{K}_{b}{\left(r-{r}_{0}\right)}^{2}+\frac{1}{2}{K}_{a}{\left(\theta -{\theta }_{0}\right)}^{2},$$

where *K*_*b*_ and *K*_*a*_ are the spring constants for the bond and angle, respectively, *r* is the bond length, *θ* is the angle value, and 0 is the corresponding equilibrium state. The CHARMM force field was used for CO_2_^59^. The OPLS-AA force field was employed for CH_4_^60^. The force field parameters for H_2_^61^, graphene^62^, and BN^63^ were taken from the references. All MD simulations were carried out using LAMMPS^64^.

### Reporting summary

Further information on research design is available in the Nature Portfolio Reporting Summary linked to this article.

## Supplementary information

Supplementary Information

Reporting Summary

## Data Availability

All data that support the findings of this study are available within the paper’s Supplementary Information or from the corresponding author upon request.
