# Supplementary material for: Pyro-layered heterostructured nanosheet membrane for hydrogen separation
Source: Nat Commun. 2023 Apr 15;14:2161. doi: 10.1038/s41467-023-37932-9 (PMC10105703; doi:10.1038/s41467-023-37932-9)
Supplement: Supplementary file 1 — Supplementary Information [file 41467_2023_37932_MOESM1_ESM.pdf]

Supplementary Information

Pyro-layered Heterostructured Nanosheet Membrane for Hydrogen Separation

Wang et al.

1. Supplementary notes

1.1. Materials

h-BN powder was purchased from Momentice USA. Urea (CH_4N_2O) was purchased from Riedel-de Haën. Chitosan (low molecular weight) and glutaraldehyde solution (50 wt. % in H_2O) were purchased from Sigma–Aldrich. All chemicals were used without further purification.

Gases of H_2, N_2, O_2, CO_2, CH_4 and Ar with a purity of 99.999 % were purchased from BOC Australia. Milli-Q water was used in the precursor solution for membrane preparation.

1.2. Characterization analysis

X-ray diffraction (XRD) analysis:

The interlayer spacings were calculated from the diffraction angles by Bragg's law

$$\lambda = 2d \bullet sin\theta \tag{1}$$

where λ=1.5406 Å for Cu Kα radiation and 2θ is the diffraction angle.

Thermogravimetric analysis (TGA):

The mass percentages of BN (without functionalized groups) and graphene in the BNG membrane were obtained by using the following equation:

$$BN\ \% + Graphene\ \% = 100\ \% \tag{2}$$

The detailed calculations of the actual mass percentage of BN are included below:

The mass loss that occurred from 20~110 °C is attributed to the absorbed moisture from the air (gray dashed line indicated), which should be excluded from the calculation of the total mass. Boron nitride has high heat resistance. The mass left after high-temperature heating in the air should be BN (see Supplementary **Fig. 19**). Thus, the mass percentage of BN in the BN/graphene system is calculated as:

$$Mass\ percentage\ of\ BN = BN/Total\ mass \times 100\ \% \tag{3}$$

Mass percentage of BN in FBN-CS precursor solution (FBN: chitosan=7: 10):

$$7/(7+10) \times 100\ \% = 41.18\ \% \tag{4}$$

Mass percentage of BN in the FBN-CS precursor membrane (FBN: chitosan=7: 10, yellow line indicated in main text **Fig. 2a**):

The moisture loss is 8.46 %. Total mass loss is approximately 84 %

$$(100\text{-}84\ \%)/(100\text{-}8.46\ \%)=17.50\ \% \tag{5}$$

Mass percentage of BN in the BNG membrane (FBN: chitosan=7: 10, orange line indicated in main text **Fig. 2a**):

The moisture loss is 4.76 %. The total mass loss is approximately 49.33 %.

$$(100\ \%\text{-}49.33\ \%)/(100\ \%\text{-}4.76\ \%)=53.20\ \% \tag{6}$$

2. Supplementary Figures

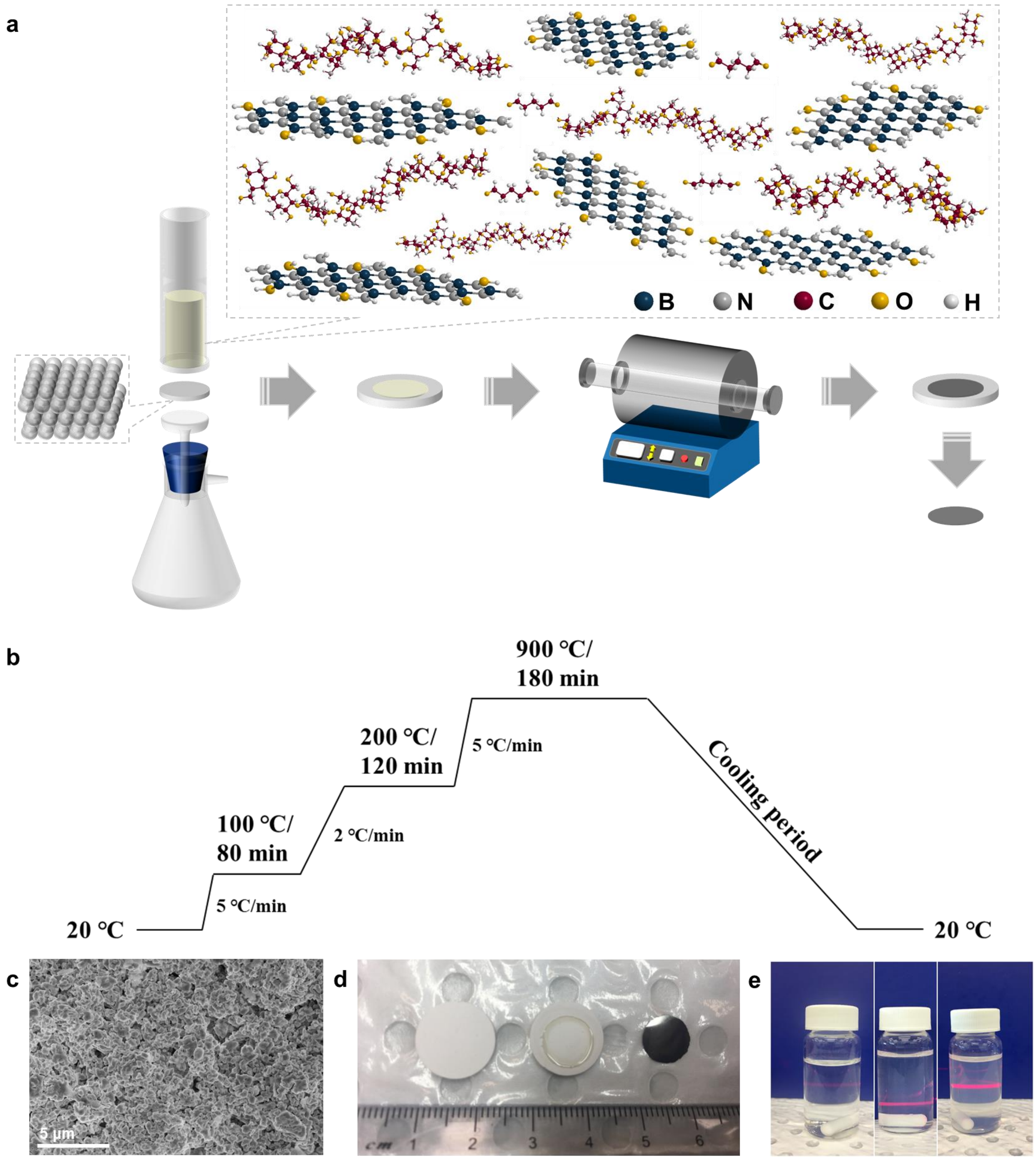

Supplementary Figure 1 BNG heterostructured membrane preparation process and (a) schematic illustration of the preparation of the FBN-chitosan precursor membrane and pyro-layering of the BNG membrane. (b) Steps involved in the membrane pyro-layering process. Ramps represent the heating period with individual heating rates. Horizontal steps represent

the dwell temperature and duration. (c) SEM surface image of the aluminum oxide substrate. (d) Digital images of the aluminum oxide substrate, precursor FBN-chitosan membrane filtered on the substrate and BNG membrane (FBN: chitosan=7:10). (e) Tyndall effect of the FBN-chitosan precursor solutions with different FBN:chitosan ratios.

Supplementary Figure 1c shows the rough surface morphology of the inhouse aluminum oxide substrate, and the irregularly shaped pores of the inhouse aluminum oxide substrate are approximately 100 nm to 500 nm. **Supplementary Figure 1d** shows digital photos of the substrate, the FBN-chitosan precursor membrane, and the as-prepared BNG membrane. The BNG membrane was peeled off from the aluminum oxide substrate. The Tyndall scattering effect in the FBN-chitosan precursor solutions illustrates a homogenous dispersion (**Supplementary Figure 1e**).

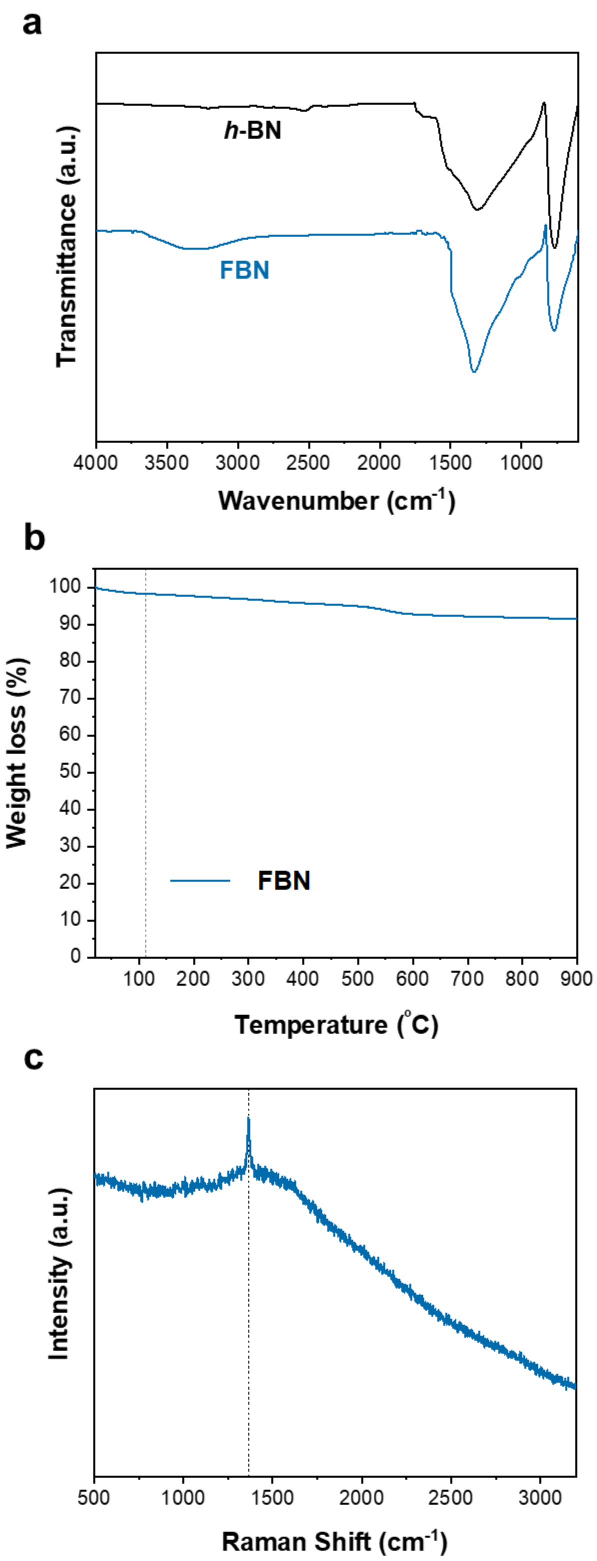

Supplementary Figure 2 Characterization of h-BN powder and FBN filtered film. (a) FTIR spectrum of the FBN film and *h*-BN powders. (b) Thermogravimetric analysis (TGA) of the FBN film. (c) Raman spectra of the FBN film.

Supplementary Figure 2a shows two distinctive bands at 1345 cm^{-1} and 775 cm^{-1} in the *h*-BN and FBN spectra, which can be ascribed to in-plane B–N stretching vibrations and out-of-plane B–N–B bending vibrations, respectively. In addition, the FBN has two additional peaks occurring at 3350 cm^{-1} and 3217 cm^{-1}, which can be ascribed to the N-H stretching vibration, representing the bonding of the functionalized groups. **Supplementary Figure 2**b shows that approximately 5 % of the functionalized groups on FBN were created after the exfoliation and functionalization process. The Raman spectra (**Supplementary Figure 2**c) show that the FBN has an outstanding BN peak at approximately 1381 cm^{-1}.

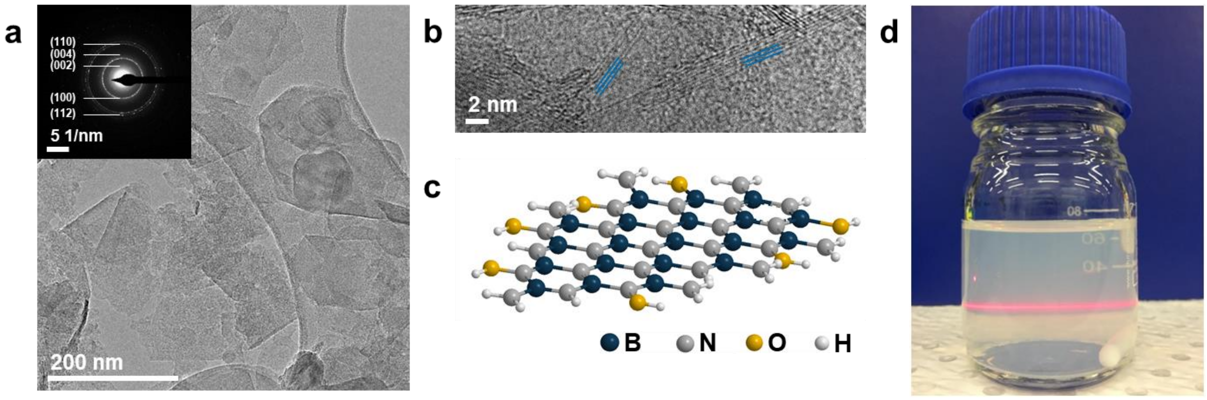

Supplementary Figure 3 Morphology and structure of exfoliated FBN. (a) TEM image of exfoliated FBN. The inset shows the selected area electron diffraction pattern of the exfoliated FBN. (b) HRTEM image of FBN with several layers. (c) Chemical structural illustration of FBN. (d) Tyndall effect of the 1 mg/mL FBN colloidal suspension.

Supplementary Figure 3a shows that the FBN has a thin and laminar morphology. The lateral size of the exfoliated FBN is relatively uniform, ranging from 80 to 150 nm. The selected area electron diffraction (SAED) image (**Supplementary Figure 3**a, inset) shows several characteristic patterns ((002), (100), (004), (110) and (112)). High-resolution TEM (HRTEM; **Supplementary Figure 3**b) shows that the interlayer spacing of FBN is 3.3 Å (blue lines indicated). In addition, the exfoliated FBNs are only a few layers thin (5-10 layers), and the thicknesses range from 2-5 nm. **Supplementary Figure 3**c shows an illustration of the chemical structure of exfoliated FBN. **Supplementary Figure 3**d shows the Tyndall effect of the prepared FBN solution. The exfoliated FBN aqueous suspension was stable and translucent.

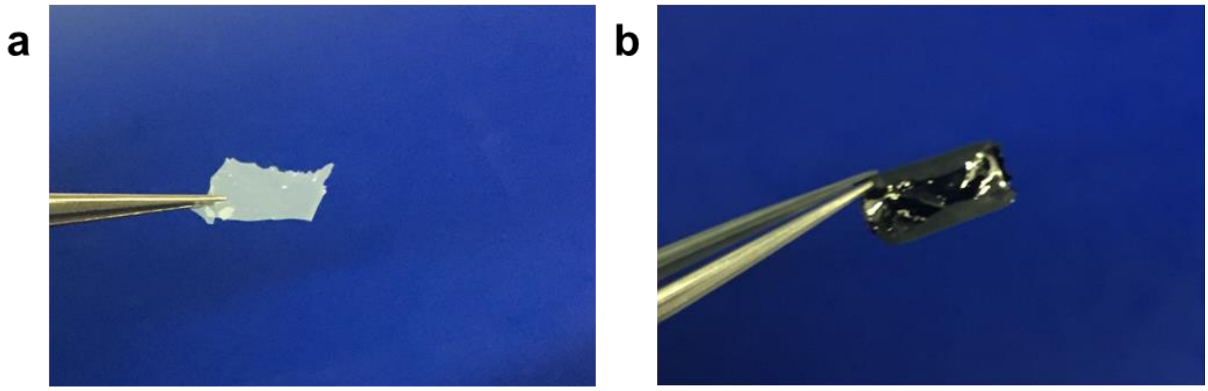

Supplementary Figure 4 Digital images of the prepared (a) pure FBN film and (b) carbonized chitosan film.

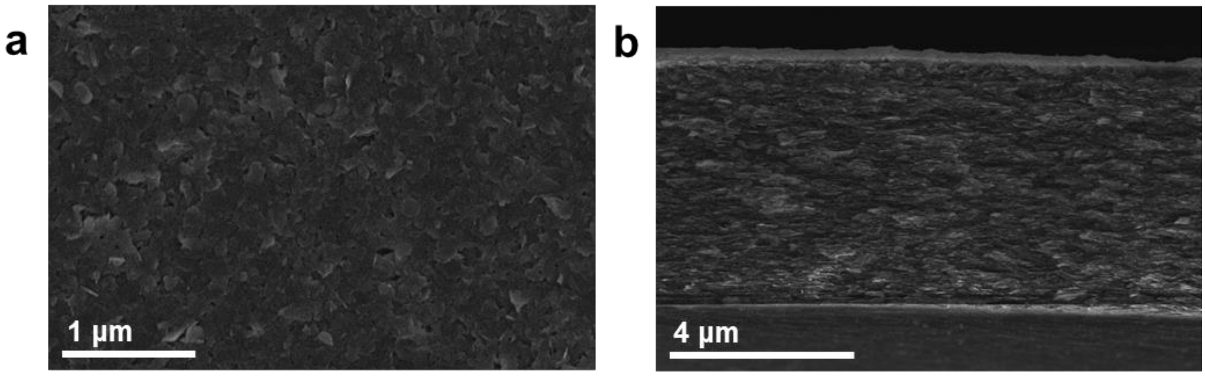

Supplementary Figure 5 SEM images of the FBN-filtered film. (a) SEM surface image of pure free-standing FBN film (peeled off from commercial AAO substrate) (b) SEM cross-sectional image of pure free-standing FBN film.

The pure compacted FBN film (**Supplementary Figure 5**a and b) shows that the lateral size of the assembled nanosheets is relatively uniform and horizontally oriented.

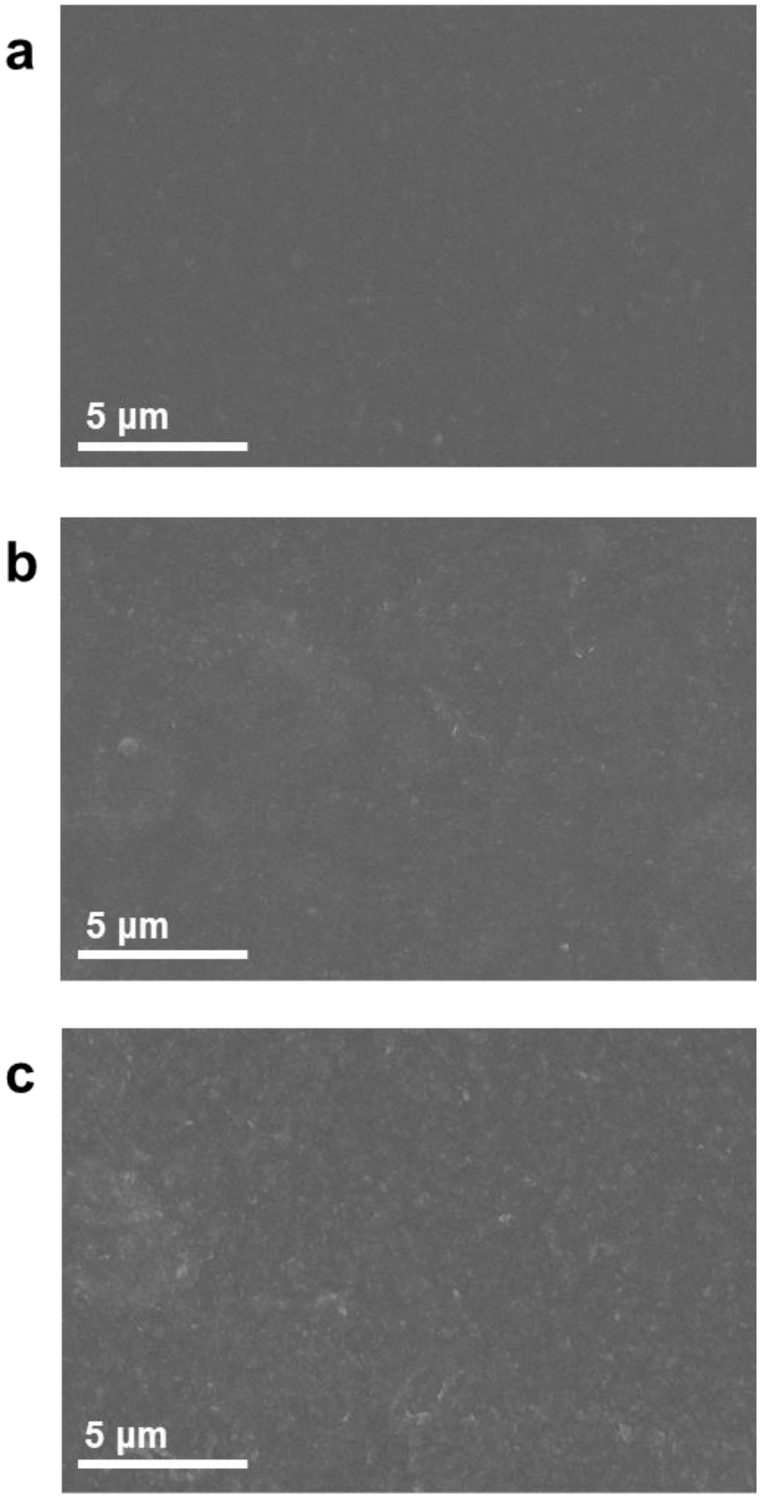

Supplementary Figure 6 SEM images of the fabricated FBN-chitosan precursor membranes (FBN: chitosan=7:25 (a), 7:10 (b) and 7:5 (c)) before high-temperature pyro-layering.

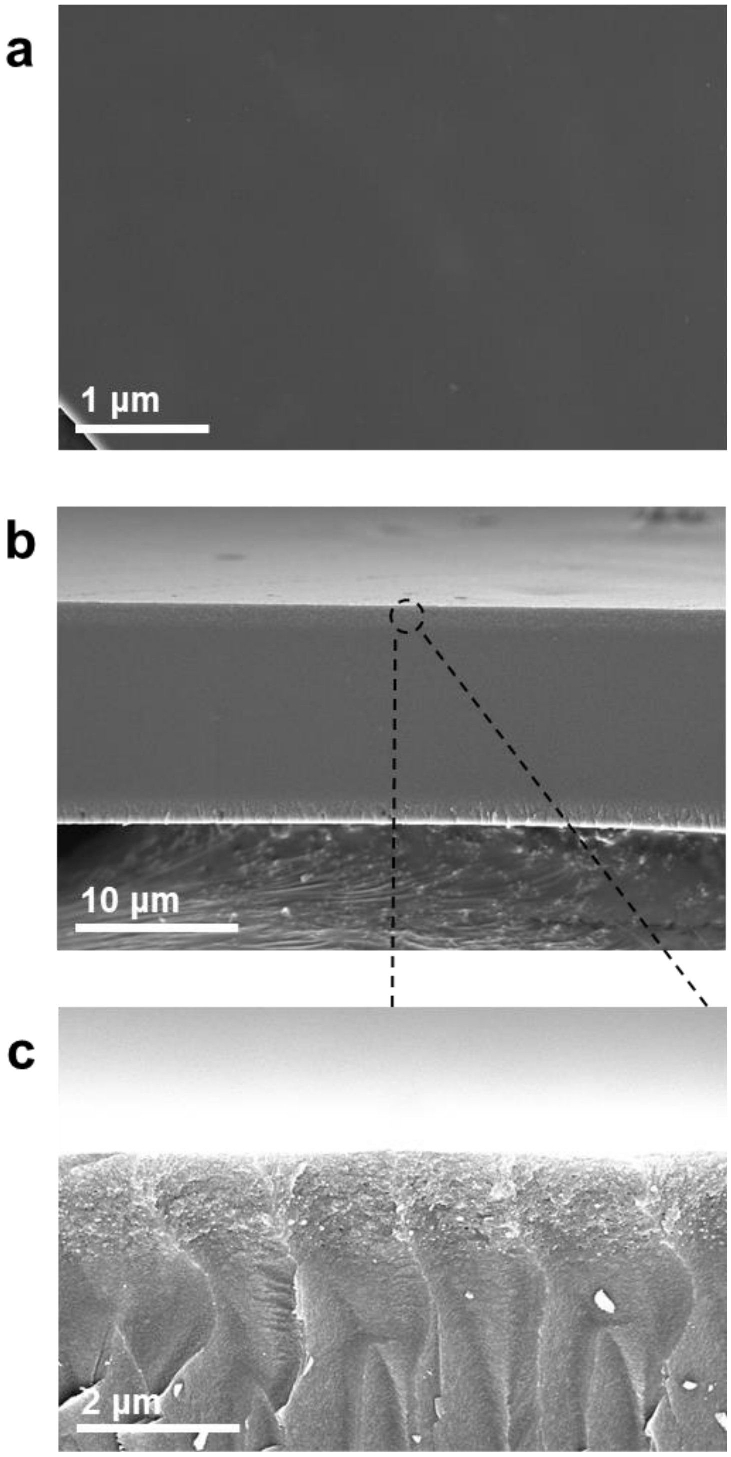

Supplementary Figure 7 SEM morphology of the pure carbonized chitosan film. (a) SEM surface image of the carbonized chitosan film. (b and c) SEM cross-sectional image of the carbonized chitosan film.

SEM images of the pure carbon film derived from chitosan show a dense morphology.

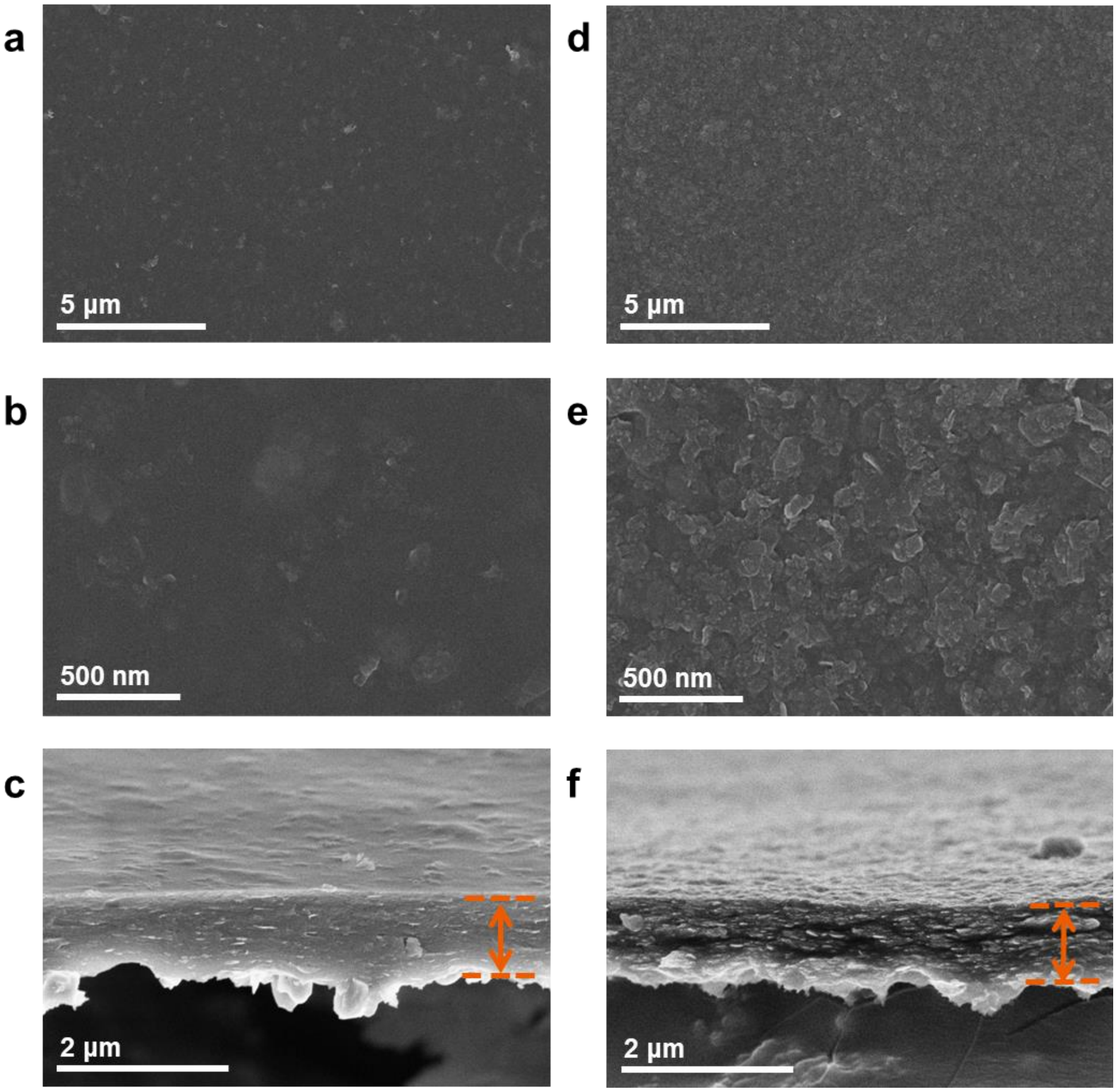

Supplementary Figure 8 SEM images of the fabricated BNG heterostructured membranes (FBN: chitosan=7:25 and 7:5). (a, b and c) SEM surface and cross-sectional images of the BNG membrane with FBN:chitosan=7:25. (d, e and f) SEM surface and cross-sectional images of the BNG membrane with FBN:chitosan=7:5.

The SEM surface images show that the BNG membranes (FBN: chitosan=7:25 and 7:5) have a surface non-defective morphology similar to that of the BNG membrane (FBN: chitosan=7:10), except for the different loadings of the embedded BN. The thicknesses of the BNG membranes (FBN: chitosan=7:25 and 7:5) are approximately 800~900 nm.

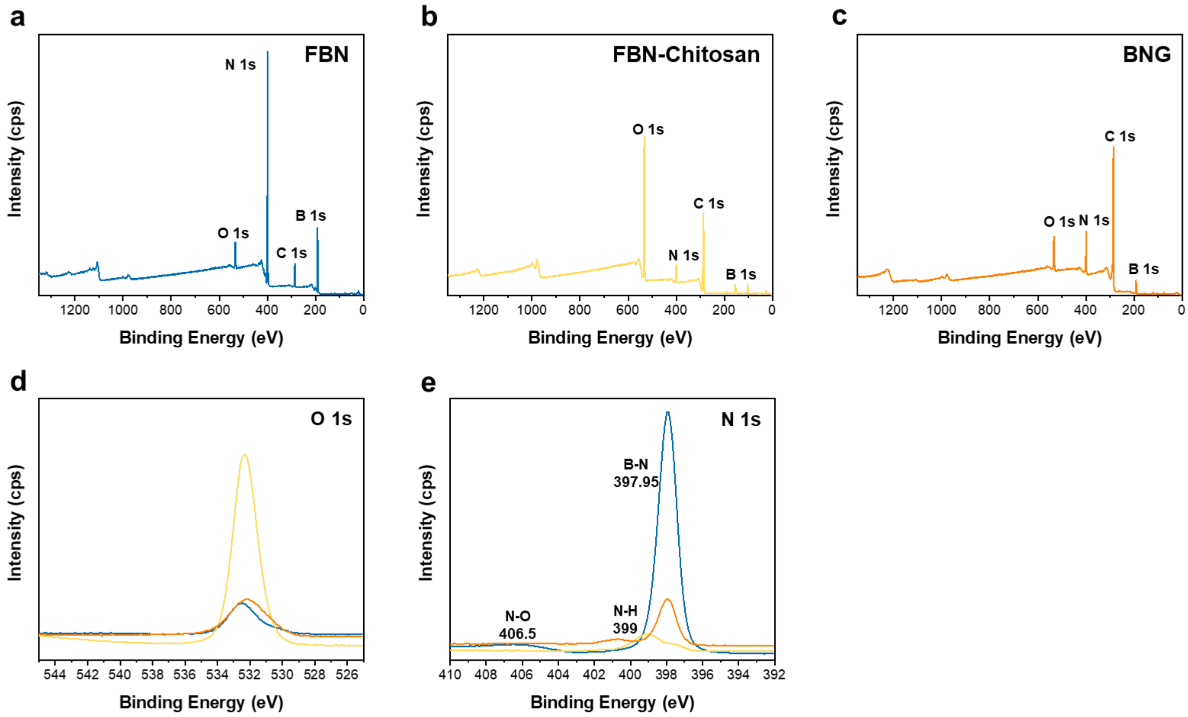

Supplementary Figure 9 XPS analysis of the chemical states of the elements on the surface of the FBN, FBN-chitosan precursor membrane (FBN: chitosan=7:10) and BNG heterostructured membrane (FBN: chitosan=7:10). (a-c) Complete survey XPS plots of FBN, the FBN-chitosan precursor membrane and the BNG membrane. (d and e) XPS spectra of O 1 s and N 1 s for samples: FBN (blue), FBN-chitosan precursor membrane (yellow) and BNG membrane (orange).

Changes in the O 1 s intensity between the FBN-chitosan precursor membrane and BNG membrane indicate the consumption of oxygen-containing groups during high-temperature pyro-layering, while the low detected O 1 s peak from FBN can be attributed to -OH groups and absorbed water or gas from the air. The C 1 s spectrum illustrates the conversion to graphitic carbon (C-C, sp^2 C) after the carbonization process.

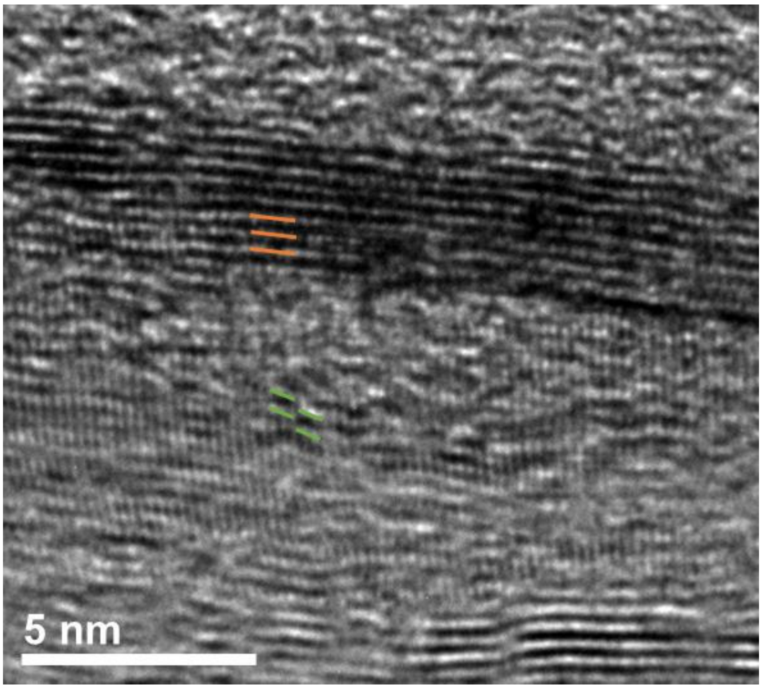

Supplementary Figure 10 HRTEM image of the BNG heterostructured membrane cross-section (FBN: chitosan=7:10). The HRTEM image reveals the embedded BN and adjoining graphene sheets. The interlayer spacing of BN and graphene nanosheets are illustrated in orange and green, respectively.

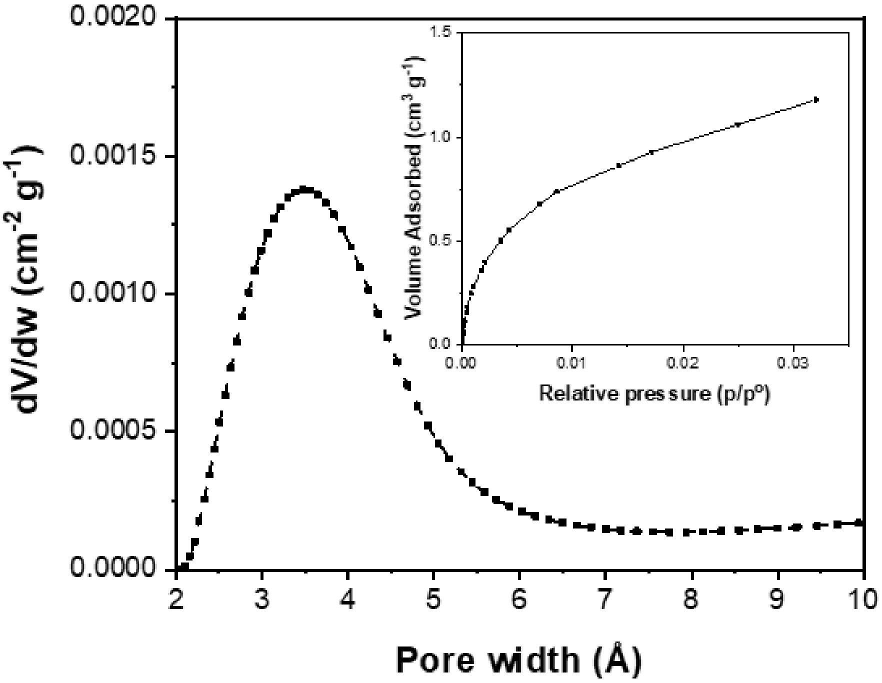

Supplementary Figure 11 Pore size distribution of the BNG membrane (FBN: chitosan=7: 10), calculated by the Horvath-Kawazon model from CO_2 physisorption at 0 °C. The inset image represents the amount of CO_2 adsorbed at low pressure at 0 °C from a pressure range of 0-1 bar.

N_2 sorption measurements were conducted, but they cannot reflect the true value of PSD. Their measured Brunauer–Emmett–Teller surface area ranges from 21 to 28 m^2/g, which is much lower than the result (100.63 m^2/g) obtained by CO_2 sorption measurement. Hence, CO_2 sorption measurements were used to show the PSD in this study.

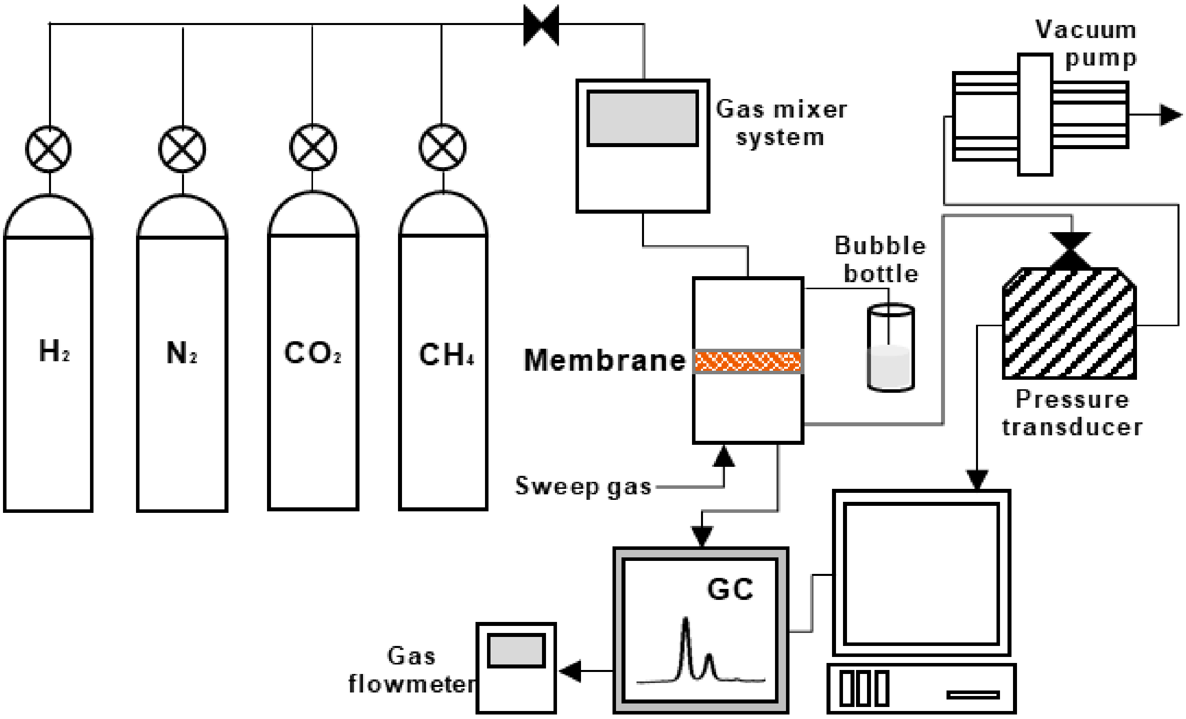

Supplementary Figure 12 Schematic of the setup for evaluating the membrane gas separation performance.

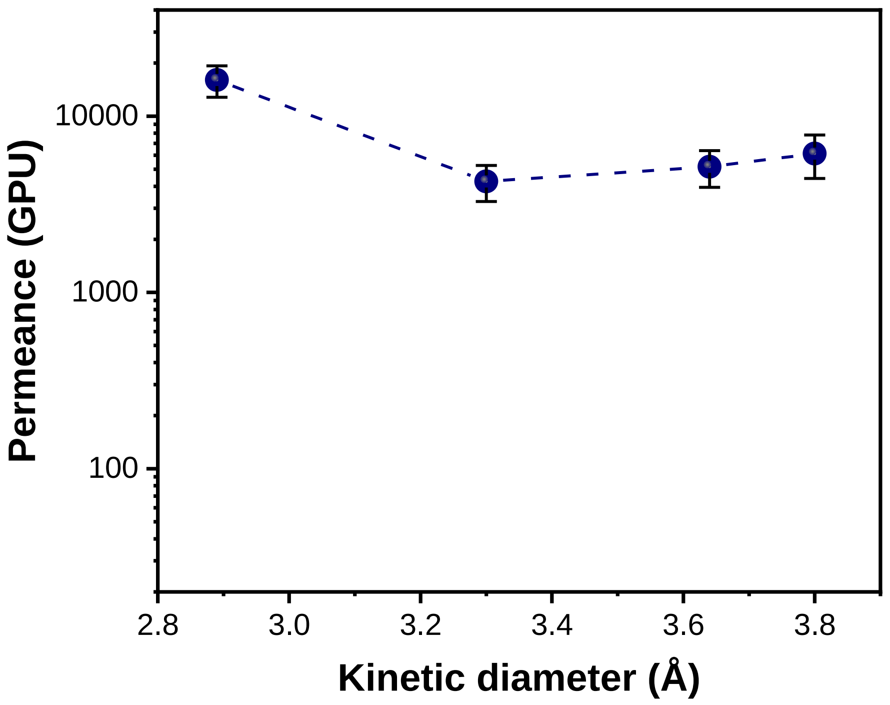

Supplementary Figure 13 Gas separation performance of the porous aluminum oxide substrate. Thickness of the aluminum oxide substrate membrane: 0.1 cm.

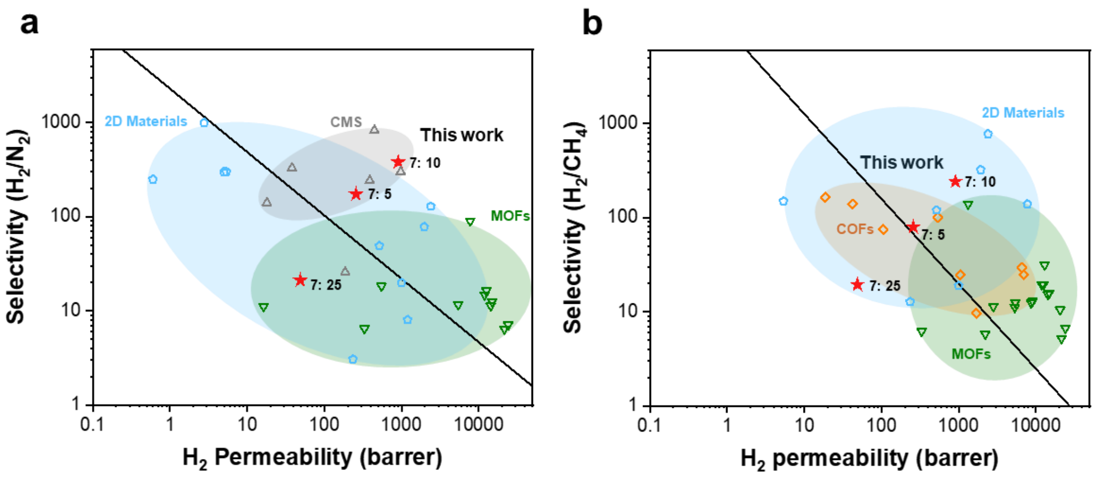

Supplementary Figure 14 Gas separation performance of our BNG heterostructured membranes compared with other molecular sieving membranes. Relationship between (a) H_2 permeability and H_2/N_2 ideal selectivity. (b) H_2 permeability and H_2/CH_4 ideal selectivity.

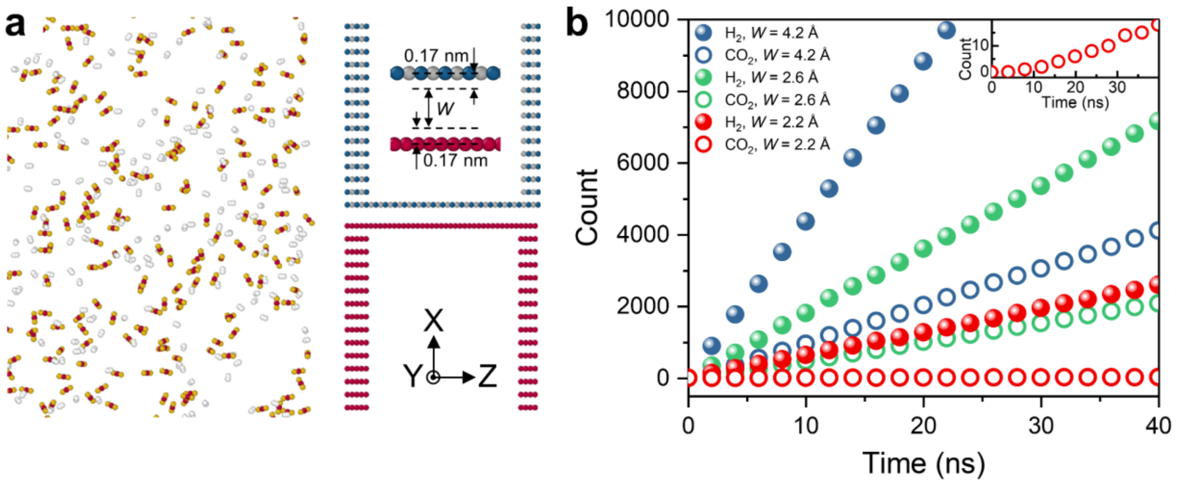

Supplementary Figure 15 (a) A snapshot of a pressure-driven flow through a BN/graphene slit-like nanochannel in the MD simulation. Inset, the illustration of channel width measurement. White: hydrogen atoms, orange: oxygen atoms, red: carbon atoms, gray: nitrogen atoms, blue: boron atoms. (b) The linear correlation between the time and the amount of gas molecules that have passed through the BN/graphene slits.

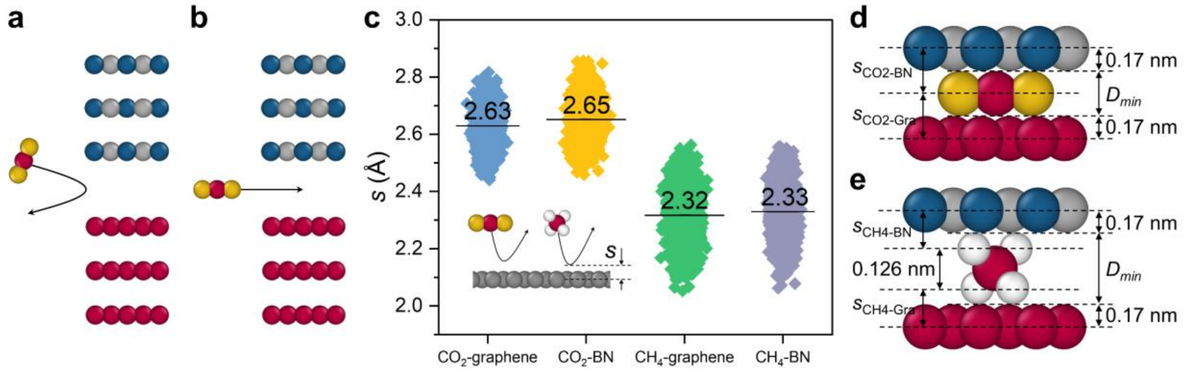

Supplementary Figure 16 Estimation of the molecular shortest dimension, D_{min}. (a and b) Orientation-determined entry failure or success for a CO_2 molecule attempting to enter a slit with the width comparable to its molecular size. (c) The shortest distance, *s*, between the gas and wall during collisions. Each dot represents a data point, and the horizontal lines show the average value from over 3000 samples for each gas-wall system. Inset, schematic of gas-wall collisions. The curved arrows denote the trajectories of gas molecules. (d and e) The illustrations of computing the molecular shortest dimension of CO_2 and CH_4. White: hydrogen atoms, orange: oxygen atoms, red: carbon atoms, gray: nitrogen atoms, blue: boron atoms.

The shortest dimension of the gas molecule is taken by considering the size of the molecule's atoms. The atomic size in MD simulations is affected by the force field and the system. In this work, the atomic size is calculated as the distance between the atomic center and the wall at contact minus half the wall's thickness (1.7 Å). We conducted 3000 collisions between the gas and wall for each of the CO_2-graphene, CO_2-BN, CH_4-graphene, and CH_4-BN systems. The shortest distance between the gas and wall was recorded for each collision and the average for each system was calculated (**Supplementary Figure 65**c). The shortest dimension of the gas molecule was calculated from the shortest distances between the gas molecule and slit walls. CO_2 was determined to have a shortest dimension of 1.87 Å (**Supplementary Figure 16**d) and CH_4 was found to have the shortest dimension of 2.51 Å (**Supplementary Figure 16**e).

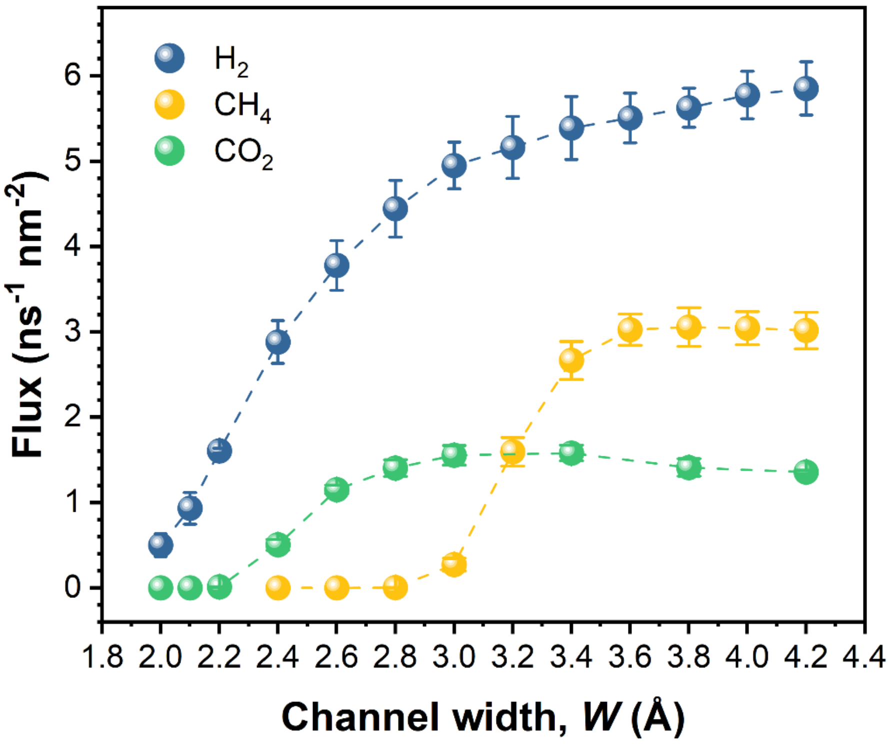

Supplementary Figure 17 Changes in the gas flux of H_2, CH_4, and CO_2 in molecular simulations as channel width varies.

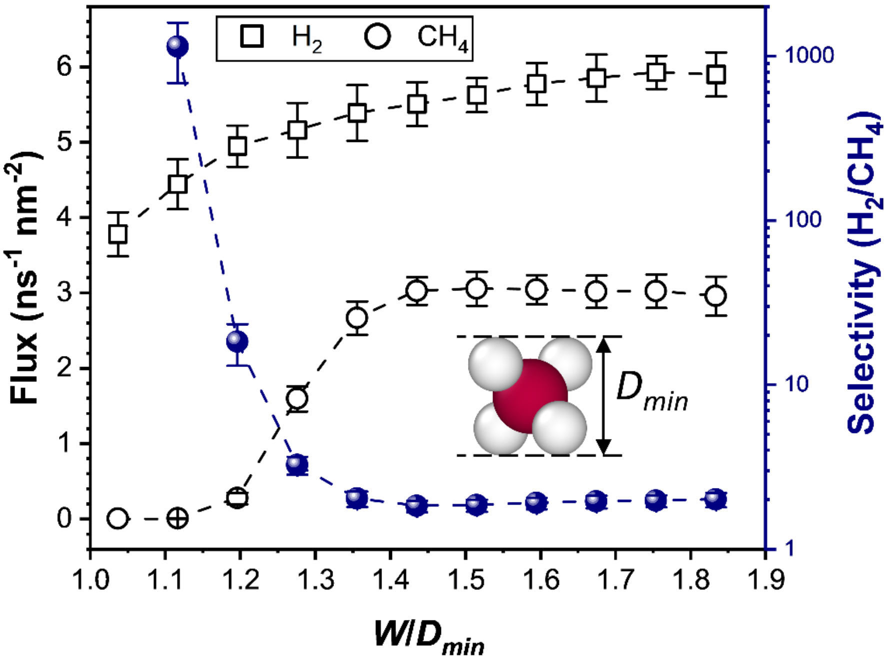

Supplementary Figure 18 The dependence of gas flux and H_2/CH_4 selectivity on the slit width relative to the shortest dimension of CH_4. Inset, the illustration of shortest dimension measurement.

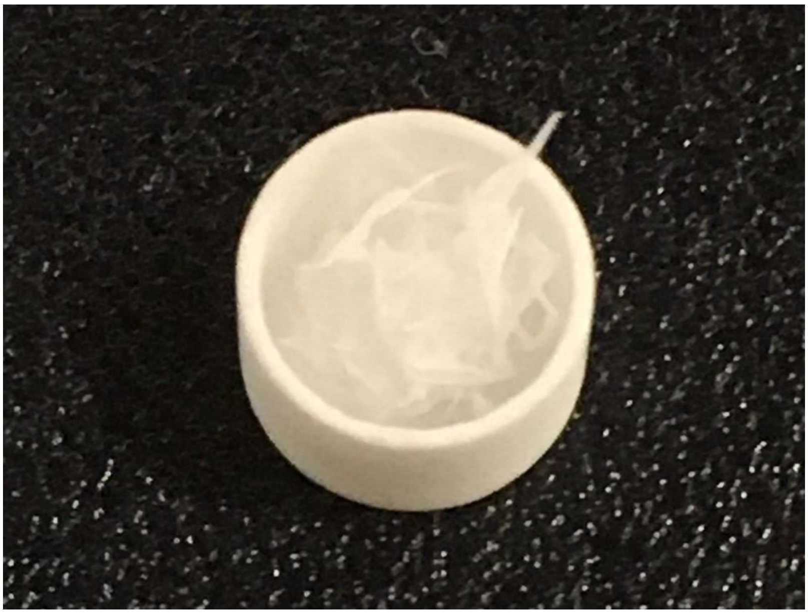

Supplementary Figure 19 White residue BN after the TGA heating test in the air (sample holder volume: 90 μL).

The BN white residue treated after the thermogravimetric analysis (TGA) test in air reveals its high heat-resisting property and proves that BN dominates the combination structure.
The TGA curve illustrates the outstanding heating stability of BN from room temperature to 900 ˚C in an air atmosphere. Based on weighting residues, the weight ratio of BN in the composite layers and functionalized groups bonded on FBN can be calculated accordingly.

3. Supplementary Tables

Supplementary Table 1 PALS results for the BNG heterostructured membrane and pure carbonized chitosan film.

	Lifetime2 (ns)	Average Pore Diamter (Å)
**BNG membrane**	0.320 ± 0.002	3.72 ± 0.14
**Pure carbonized chitosan**	0.343 ± 0.002	4.35 ± 0.10

Supplementary Table 2 Comparison of the gas permeability, permeance and selectivity (H_2/CO_2) of the BNG heterostructured membranes with those of other molecular sieving membranes (literature data). Carbon molecular sieving (CMS) membranes are presented as CMS.

	Membrane materials	Permeability (Barrer)	Permeance (GPU)	Type of analysis	H_2/CO_2	Ref.
**CMS**	CMS-1	1065	118.3	Single	14.5	1
	CMS-2	228.4	1827.2	Single	8	2
	CMS-3	54	N.A.	Single	80	3
	CHFM-550	1400.3	466.8	Single	11.1	4
	CHFM-700	773.3	257.8	Single	49.5	
	CHFM-850	444.6	148.2	Single	83.9	
**MOFs**	ZIF-7	331	227.2	Mixed	6.5	5
	ZIF-7	54.2	27.1	Mixed	8.4	6
	ZIF-8	3536.4	294.7	Mixed	6	7
	ZIF-8	2534.8	1267.4	Mixed	3.3	8
	ZIF-8	5411	180.4	Single	4.4	9
	ZIF-22[a]	24130	603.3	Single	8.5	10
	ZIF-22[b]	21502	537.6	Mixed	7.8	
	ZIF-90[a]	14932	746.6	Single	7.2	11
	ZIF-90[b]	14335	716.8	Mixed	7.4	
	ZIF-90[a]	12543	627.2	Single	15.7	12
	ZIF-90[b]	11946	597.3	Mixed	15.2	
	HKUST-1	176844	2947.4	Mixed	6.8	13
	NH2-MIL-53(Al)	87759	5850.6	Mixed	30.9	14
	MOF JUC-150	549	18.3	Single	38.7	15
	MOF JUC-150	452.4	15.1	Mixed	30.2	
	Amine-Mg-MOF-74	2240	224	Mixed	28	16
	MIL-96 (Al)	12260.8	1532.6	Mixed	8.8	17
	Zn2(Bim)3	19.2	1918.7	Mixed	128.4	18
**COFs**	TpPa-1(40)@PBI-BuI	18.6	0.3	Mixed	165.5	19
	TpBD(50)@PBI-BuI	42.1	0.6	Mixed	139.7	
	NUS-2@PBI	3	0.1	Mixed	18.8	20
	[COF-300]-[Zn2(bdc)2(dabco)]	130368	1344	Mixed	12.6	21
	[COF-300]-[ZIF-8]	105520	1055.2	Mixed	13.5	
	COF-300	107520	2688	Mixed	6	
	COF-LZU1	1842.1	3684.2	Mixed	6	22
	ACOF-1	1211.3	2018.9	Mixed	14.1	
	COF-LZU1–ACOF-1	660.2	660.2	Mixed	24.2	
	TpPa-1-30/GO-10	943.5	3144.9	Mixed	25.6	23
	Vertically aligned COF-LZU1	7309.6	3654.8	Mixed	31.6	24
	Vertically aligned TFB-BD	7604.4	3802.2	Mixed	25.6	

	TpEBr@TpPa-SO_3NA	105.2	2566.1	Mixed	22.6	25
**2D Materials**	ZIF-8/GO (LBL)	7761.2	388.1	Mixed	14.9	26
	GO	0.6	333.3	Mixed	2100	27
	GO	2.8	307.8	Mixed	3400	
	GO	5	277.2	Mixed	2300	
	GO (dry)	2.5	35	Mixed	30	28
	EFDA-GO	1200	1200	Mixed	29	29
	TU-GOF-1	234.4	2604.1	Mixed	8.5	30
	TU-GOF-3	514.9	627.9	Mixed	100	
	TU-GOF-5	999.2	322.3	Mixed	32	
	MXene 20 nm	31.2	1561.5	Mixed	27	31
	MXene[a]	2402.3	1201.2	Single	238	32
	MXene[b]	2226.6	1113.3	Mixed	167	
	MoS_2	240	N.A.	Mixed	8.5	33
	BNG (7:25)	49.51	58.3	Single	33.6	This work
	BNG (7:10)	920.39	1150.5	Single	388.8	
	BNG (7:5)	261.21	290.2	Single	222.1	

Supplementary Table 3 Comparison of the gas permeability, permeance and selectivity (H_2/N_2) of the BNG heterostructured membranes with those of other molecular sieving membranes (literature data).

	Membrane materials	Permeability (Barrer)	Permeance (GPU)	Type of analysis	H_2/N_2	Ref.
**CMS**	CMS-1	38.1	605	Single	331	34
	CMS-2	18	55.9	Single	141.2	35
	CMS-3	974	9740	Single	302	36
	CMS-4	186.3	~ 186.3	Single	25.8	37
	CMS-5	388.3	107.9	Single	243	38
	CHFM-850	444.6	148.2	Single	829	4
**MOFs**	ZIF-7	331	220.7	Mixed	6.5	5
	ZIF-8	5411	180.4	Mixed	11.6	9
	ZIF-22[a]	24130	603.3	Single	7.1	10
	ZIF-22[b]	21502	537.6	Mixed	6.4	
	ZIF-90[a]	14932	746.6	Single	12.6	11
	ZIF-90[b]	14335	716.8	Mixed	11.3	
	ZIF-90[a]	12543	627.2	Single	16.4	12
	ZIF-90[b]	11946	597.3	Mixed	14.8	
	MOF JUC-150	549	18.3	Mixed	17.1	15
	ZIF-8/GO (seeding layer)	16.3	163	Mixed	11.1	25
**COFs**	TpEBr@TpPa-SO_3NA	105.2	2566.1	Mixed	40.5	34
**2D Materials**	ZIF-8/GO (LBL)	7761.2	388.1	Mixed	90.5	26
	GO[a]	5.4	298.9	Mixed	300	27
	GO[b] (CA)	0.6	333.3	Mixed	250	
	GO[b]	2.77	307.8	Mixed	1000	
	GO[b]	5	277.2	Mixed	300	
	EFDA-GO	1200	1200	Mixed	8	29
	TU-GOF-1	234.4	2604.1	Mixed	3	30
	TU-GOF-3	514.9	627.9	Mixed	49	
	TU-GOF-5	999.2	322.3	Mixed	19.8	
	MXene[a]	2402.3	1201.2	Single	129	32
	MXene[b]	1975.9	988	Mixed	78	
	BNG (7:25)	49.51	58.3	Single	20.7	This work
	BNG (7:10)	920.39	1150.5	Single	375.1	
	BNG (7:5)	261.21	290.2	Single	171.1	

Supplementary Table 4 Comparison of the gas permeability, permeance and selectivity (H_2/CH_4) of the BNG heterostructured membranes with molecular sieving membranes (literature data).

	Membrane materials	Permeability (Barrer)	Permeance(GPU)	Type of analysis	H_2/CH_4	Ref.
**MOFs**	ZIF-7	331	220.7	Mixed	6.2	5
	ZIF-8	5411	180.4	Mixed	12.6	9
	ZIF-8	20632.5	589.5	Mixed	10.5	39
	ZIF-8	12966	648.3	Mixed	31.5	40
	ZIF-8	9078	4539	Mixed	13	41
	ZIF-8	2811.6	468.6	Mixed	11.4	42
	ZIF-8	8666	433.3	Mixed	12.5	43
	ZIF-22[a]	24130	603.3	Single	6.7	10
	ZIF-22[b]	21502	537.6	Mixed	5.2	
	ZIF-90[a]	14932	746.6	Single	15.9	11
	ZIF-90[b]	14335	716.8	Mixed	15.3	
	ZIF-90[a]	12543	627.2	Single	19.4	12
	ZIF-90[b]	11946	597.3	Mixed	19.4	
	MOF JUC-150	549	5.2	Mixed	5.2	5
	HKUST-1	176844	2947.4	Mixed	6	13
	NH2-MIL-53(Al)	67068	4471.2	Mixed	207	14
	Amine-Mg-MOF-74	2211	221.1	Mixed	5.8	16
	2abIm-VPLT-RTD-ZIF-8	1326.3	442.1	Mixed	140	44
**COFs**	TpPa-1(40)@PBI-BuI	18.7	0.3	Mixed	165.5	19
	TpBD(50)@PBI-BuI	42.1	0.6	Mixed	139.7	
	COF-LZU1	1694.8	3389.5	Mixed	9.7	22
	ACOF-1	1046.9	1744.9	Mixed	24.7	
	COF-LZU1–ACOF-1	536.4	536.4	Mixed	100.2	
	Vertically aligned COF-LZU1	6602.2	3301.1	Mixed	29.5	24
	Vertically aligned TFB-BD	6955.8	3477.9	Mixed	24.9	
	TpEBr@TpPa-SO_3NA	105.2	2566.1	Mixed	75.2	34
**2D Materials**	GO[a]	5.4	298.9	Single	~150	27
	TU-GOF-1	234.4	2604.1	Mixed	12.8	30
	TU-GOF-3	514.9	627.9	Mixed	120	
	TU-GOF-5	999.2	322.3	Mixed	18.9	
	ZIF-8/GO (LBL)	7761.2	388	Mixed	139.1	26
	MXene[a]	2402.3	1201.2	Single	780	32
	MXene[b]	1930.6	965.3	Mixed	324	
	BNG (7:25)	49.51	58.3	Single	19.1	This work
	BNG (7:10)	920.39	1150.5	Single	239.4	
	BNG (7:5)	261.21	290.2	Single	78.3	

Supplementary Table 5 Single gas permeability and selectivity of BNG heterostructured membranes with different FBN:chitosan ratios. Mixed gas selectivity (H_2/CO_2) of BNG membranes.

	Single gas permeability (Barrer)				Ideal selectivity					Mixed gas permeability (Barrer)		Mixed gas permeance (GPU)		Mixed selectivity
**FBN: Chitosan**	**H_2**	**N_2**	**CO_2**	**CH_4**	**H_2/N_2**	**H_2/CO_2**	**H_2/CH_4**	**CO_2/N_2**	**CO_2/CH_4**	**H_2**	**CO_2**	**H_2**	**CO_2**	**H_2/CO_2**
**7:25**	49.5	2.4	1.5	2.6	20.7	33.6	19.1	0.7	0.5	46.2	2.7	54.4	3.2	17.2
**7:10**	920.4	2.5	2.4	3.8	375.1	388.8	239.4	1.0	0.6	849.0	2.9	1061.3	3.6	289.5
**7:5**	261.2	1.6	1.3	3.4	171.1	222.1	78.3	0.9	0.4	224.5	1.5	249.4	1.7	151.4

Supplementary References

1. Sá, S., Sousa, J. M. & Mendes, A. Steam reforming of methanol over a $CuO/ZnO/Al_2O_3$ catalyst part II: A carbon membrane reactor. *Chem. Eng. Sci.* **66**, 5523–5530 (2011).
2. Richter, H. *et al.* High-Flux Carbon Molecular Sieve Membranes for Gas Separation. *Angew. Chemie Int. Ed.* **56**, 7760–7763 (2017).
3. Omidvar, M. *et al.* Unexpectedly Strong Size-Sieving Ability in Carbonized Polybenzimidazole for Membrane H_2/CO_2 Separation. *ACS Appl. Mater. Interfaces* **11**, 47365–47372 (2019).
4. Lei, L. *et al.* Carbon hollow fiber membranes for a molecular sieve with precise-cutoff ultramicropores for superior hydrogen separation. *Nat. Commun.* **12**, 268 (2021).
5. Li, Y.-S. *et al.* Molecular Sieve Membrane: Supported Metal-Organic Framework with High Hydrogen Selectivity. *Angew. Chemie* **122**, 558–561 (2010).
6. Li, B. Y., Bux, H., Feldhoff, A., Li, G. & Yang, W. Controllable Synthesis of Metal–Organic Frameworks : From MOF Nanorods to Oriented MOF Membranes. *Adv. Mater.* **22**, 3322–3326 (2010).
7. Bux, H. *et al.* Oriented Zeolitic Imidazolate Framework-8 Membrane with Sharp H_2/C_3H_8 Molecular Sieve Separation. *Chem. Mater.* **23**, 2262–2269 (2011).
8. Huang, K., Dong, Z., Li, Q. & Jin, and W. Growth of a ZIF-8 membrane on the inner-surface of a ceramic hollow fiber via cycling precursors. *Chem. Commun.* **49**, 10326–10328 (2013).
9. Bux, H., Liang, F., Li, Y., Cravillon, J. & Wiebcke, Michael Caro, J. Zeolitic Imidazolate Framework Membrane with Molecular Sieving Properties by Microwave-Assisted Solvothermal Synthesis. *J. Am. Chem. Soc.* **21**, 4920–4924 (2009).
10. Huang, A., Bux, H., Steinbach, F. & Caro, J. Molecular-Sieve Membrane with Hydrogen Permselectivity: ZIF-22 in LTA Topology Prepared with 3-Aminopropyltriethoxysilane as Covalent Linker. *Angew. Chemie* **122**, 5078–5081 (2010).
11. Huang, A., Dou, W. & Caro, J. Steam-stable zeolitic imidazolate framework ZIF-90 membrane with hydrogen selectivity through covalent functionalization. *J. Am. Chem. Soc.* **132**, 15562–15564 (2010).
12. Huang, A. & Caro, J. Covalent Post-Functionalization of Zeolitic Imidazolate Framework ZIF-90 Membrane for Enhanced Hydrogen Selectivity. *Angew. Chemie Int. Ed.* **50**, 4979–4982 (2011).

13. Guo, H., Zhu, G., Hewitt, I. J. & Qiu, S. "Twin Copper Source" Growth of Metal-Organic Framework Membrane : $Cu_3(BTC)_2$ with High Permeability and Selectivity for Recycling H_2. *J. Am. Chem. Soc.* **131**, 1646–1647 (2009).
14. Zhang, F. *et al.* Hydrogen Selective NH2-MIL-53 Al MOF Membranes with High Permeability. *Adv. Funct. Mater.* **22**, 3583–2590 (2012).
15. Kang, Z. *et al.* Highly selective sieving of small gas molecules by using an ultra-microporous metal-organic framework membrane. *Energy Environ. Sci.* **7**, 4053–4060 (2014).
16. Wang, N., Mundstock, A., Liu, Y., Huang, A. & Caro, J. Amine-modified Mg-MOF-74/ CPO-27-Mg membrane with enhanced H_2/CO_2 separation. *Chem. Eng. Sci.* **124**, 27–36 (2015).
17. Knebel, A. *et al.* Comparative Study of MIL-96 (Al) as Continuous Metal−Organic Frameworks Layer and Mixed-Matrix Membrane. *ACS Appl. Mater. Interfaces* **8**, 7536–7544 (2016).
18. Peng, Y., Li, Y., Ban, Y. & Yang, W. Two-Dimensional Metal Organic Framework Nanosheets for Membrane-Based Gas Separation. *Angew. Chemie* **129**, 9889–9893 (2017).
19. Biswal, B. P., Chaudhari, H. D., Banerjee, R. & Kharul, U. K. Chemically Stable Covalent Organic Framework COF -Polybenzimidazole Hybrid Membranes: Enhanced Gas Separation through Pore Modulation. *Chem. A Eur. J. Commun.* **22**, 4695–4699 (2016).
20. Kang, Z. *et al.* Mixed Matrix Membranes (MMMs) Comprising Exfoliated 2D Covalent Organic Frameworks (COFs) for Efficient CO_2 Separation. *Chem. Mater.* **28**, 1277–1285 (2016).
21. Fu, J. *et al.* Fabrication of COF-MOF Composite Membranes and Their Highly Selective Separation of H_2/CO_2. *J. Am. Chem. Soc.* **138**, 7673–7680 (2016).
22. Fan, H. *et al.* Covalent Organic Framework−Covalent Organic Framework Bilayer Membranes for Highly Selective Gas Separation. *J. Am. Chem. Soc.* **140**, 10094–10098 (2018).
23. Tang, Y. *et al.* Covalent organic frameworks combined with graphene oxide to fabricate membranes for H_2/CO_2 separation. *Sep. Purif. Technol.* **223**, 10–16 (2019).
24. Fan, H. *et al.* High-Flux Vertically Aligned 2D Covalent Organic Framework Membrane with Enhanced Hydrogen Separation. *J. Am. Chem. Soc.* **142**, 6872–6877 (2020).

25. Ying, Y. *et al.* Ultrathin Two-Dimensional Membranes Assembled by Ionic Covalent Organic Nanosheets with Reduced Apertures for Gas Separation. *J. Am. Chem. Soc.* **142**, 4472–4480 (2020).
26. Huang, A., Liu, Q., Wang, N. & Zhu, Y. Bicontinuous Zeolitic Imidazolate Framework ZIF-8@GO Membrane with Enhanced Hydrogen Selectivity. *J. Am. Chem. Soc.* **136**, 14686–14689 (2014).
27. Li, H. *et al.* Ultrathin, Molecular-Sieving Graphene Oxide Membranes for Selective Hydrogen Separation. *Science* **342**, 95–99 (2013).
28. Kim, H. W. *et al.* Selective Gas Transport Through Few-Layered Graphene and Graphene Oxide Membranes. *Science* **342**, 91–96 (2013).
29. Shen, J. *et al.* Subnanometer Two-Dimensional Graphene Oxide Channels for Ultrafast Gas Sieving. *ACS Nano* **10**, 3398–3409 (2016).
30. Yang, J. *et al.* Self-Assembly of Thiourea-Crosslinked Graphene Oxide Framework Membranes toward Separation of Small Molecules. *Adv. Mater.* **30**, 1705775 (2018).
31. Shen, J. *et al.* 2D MXene Nanofilms with Tunable Gas Transport Channels. *Adv. Funct. Mater.* **28**, 1801511 (2018).
32. Ding, L. *et al.* MXene molecular sieving membranes for highly efficient gas separation. *Nat. Commun.* **9**, 155 (2018).
33. Achari, A., Sahana, S. & Eswaramoorthy, M. High performance MoS_2 membranes: Effects of thermally driven phase transition on CO_2 separation efficiency. *Energy Environ. Sci.* **9**, 1224–1228 (2016).
34. Shiflett, M. B. & Foley, H. C. Ultrasonic Deposition of High-Selectivity Nanoporous Carbon Membranes. *Science* **285**, 1902–1905 (1999).
35. Hou, J. *et al.* Carbon Nanotube Networks as Nanosca ff olds for Fabricating Ultrathin Carbon Molecular Sieve Membranes. *ACS Appl. Mater. Interfaces* **10**, 20182–20188 (2018).
36. Ogieglo, W., Puspasari, T., Ma, X. & Pinnau, I. Sub-100 nm carbon molecular sieve membranes from a polymer of intrinsic microporosity precursor : Physical aging and near-equilibrium gas separation properties. *J. Memb. Sci.* **597**, 117752 (2020).
37. Ogieglo, W. *et al.* Thin Composite Carbon Molecular Sieve Membranes from a Polymer of Intrinsic Microporosity Precursor. *Appl. Mater. Interfaces* **11**, 18770–18781 (2019).
38. Tseng, H. *et al.* Enhanced H_2/CH_4 and H_2/CO_2 separation by carbon molecular sieve membrane coated on titania modified alumina support : Effects of TiO_2 intermediate layer preparation variables on interfacial adhesion. *J. Memb. Sci.* **510**, 391–404 (2016).

39. Cacho-bailo, F., Seoane, B., Téllez, C. & Coronas, J. ZIF-8 continuous membrane on porous polysulfone for hydrogen separation. *J. Memb. Sci.* **464**, 119–126 (2014).
40. Liu, Q., Wang, N. & Huang, A. Bio-Inspired Polydopamine: A Versatile and Powerful Platform for Covalent Synthesis of Molecular Sieve Membranes. *J. Am. Chem. Soc.* **135**, 17679–17682 (2013).
41. Pan, Y., Wang, B. & Lai, Z. Synthesis of ceramic hollow fiber supported zeolitic imidazolate framework-8 (ZIF-8) membranes with high hydrogen permeability. *J. Memb. Sci.* **421–422**, 292–298 (2012).
42. Zhang, X. *et al.* New Membrane Architecture with High Performance ZIF-8. *Chem. Mater.* **26**, 1975–1981 (2014).
43. Liu, Y., Wang, N., Pan, J. H., Steinbach, F. & Caro, J. In Situ Synthesis of MOF Membranes on ZnAl-CO_3 LDH Buffer Layer- Modified Substrates. *J. Am. Chem. Soc.* **136**, 14353–14356 (2014).
44. Eum, K. *et al.* ZIF-8 Membrane Separation Performance Tuning by Vapor Phase Ligand Treatment. *Angew. Chemie* **131**, 16542–16546 (2019).
